# Quality of life and owner attitude to dog overweight and obesity in Thailand and the Netherlands

**DOI:** 10.1186/s12917-018-1531-z

**Published:** 2018-07-09

**Authors:** Nienke Endenburg, Sirikul Soontararak, Chalermpol Charoensuk, Hein A. van Lith

**Affiliations:** 1 Division Human-Animal Relations, Department of Animals in Science and Society, and Animal Behaviour Clinic, Faculty of Veterinary Medicine, Utrecht University, Yalelaan 2, PO Box 80166, 3508 TD Utrecht, The Netherlands; 2 Division of Animal Welfare & Laboratory Animal Science, Department of Animals in Science and Society, Faculty of Veterinary Medicine, Utrecht University, Utrecht, The Netherlands; 3 Department of Companion Animal Clinical Sciences, Faculty of Veterinary Medicine, Kasetsart University, Bangkok, Thailand; 4 Presenr address: Department of Clinical Sciences, College of Veterinary Medicine & Biomedical Sciences, Colorado State University, Denver, USA; 5 Brain Center Rudolf Magnus, University Medical Center Utrecht, Utrecht, The Netherlands

**Keywords:** Body condition score (BCS), Cultural influence, Dogs, Exercise, Feeding, Obesity, Overweight, Quality of Life (QoL)

## Abstract

**Background:**

This study investigated whether the body condition score (BCS) and/or culture influences the quality of life (QoL) of dogs, as evaluated by the owner, and whether the BCS is influenced by feeding and exercise and its owner’s culture. To this end, a questionnaire was administered to 355 selected dog owners (Thai and Dutch). Their dogs had a BCS of 3 (normal weight), 4 (overweight) or 5 (obese) but no other physical problems. Instead of using Likert scales, continuous scales were used. Further, data for the questionnaire items were transformed using an integrated z-score methodology.

**Results:**

The magnitude of factor loadings was similar to that reported in a previous study, indicating that the questionnaire is not culture specific. QoL scores for general sickness were significantly higher (worse) in dogs with a higher BCS. Thus even though the dogs were apparently healthy, the BCS influenced the perceived QoL of the dog. Immobility was seen more often in dogs with a higher (poorer) BCS than in dogs with a lower (better) BCS; however, there was no clear relationship between immobility and total activity. The higher the BCS, the less owners felt in control of feeding and exercise. The BCS was higher in the dogs of owners who did not like to exercise. The Thai dogs showed more separation-related behaviour problems when their owner left home than did the Dutch dogs.

**Conclusions:**

The QoL of overweight and obese dogs is mainly influenced by the dog’s physical status. The owners of dogs with a high BCS have less perceived control over feeding and exercise. Our findings indicate that owner attitudes and beliefs essentially cause obesity as a result of a lack of knowledge and perceived control.

**Electronic supplementary material:**

The online version of this article (10.1186/s12917-018-1531-z) contains supplementary material, which is available to authorized users.

## Background

Overweight is an increasingly common problem in humans and their pets [1, 2]. New standards have been set for dogs, with obesity being defined as a weight greater than 15% of ideal [3]; standards that have consequences for health and wellbeing [4, 5]. Up to 40% of dogs in developed countries are overweight [6]. The most common cause of overweight is an energy intake exceeding energy expenditure. Several risk factors for overweight have been identified, such as breed/genetic background [7], neuter status [8], orthopaedic diseases (and thus decreased activity) [9], type of diet [10], change of lifestyle [11], body condition score (BCS) of the dog as judged by its owner [10], and owner socioeconomic status [9, 11].

Overweight and obesity are a growing problem in distinct companion dog populations and can cause discomfort and disease, and/or reducing the quality of life (QoL) of affected dogs [12]. Obesity can promote the development of a number of diseases that reduce the animal’s QoL and life span [13]. The general concept of QoL takes into account the physical, mental, and social needs of the individual, and meeting these needs is considered to reflect a positive status. Therefore, QoL assessment can be used to assess improvements in conditions that adversely affect QoL, such as prolonged discomfort and disease.

In humans, the perception of health and illness, and hence QoL, are culturally determined [14] and thereby influence the relative importance of factors such as affection, satisfaction, and acceptance, factors that are in turn influenced by lifestyle, past experience, and social consensus [15]. A person’s beliefs and attitudes to overweight, obesity, eating behaviour, and exercise are also culturally determined [16]. For example, many cultures believe that human overweight reflects wealth, prosperity, and a high status [16, 17]. However, it is not clear whether cultural beliefs influence how owners perceive the QoL of their pets. Apart from cultural belief influences, climate probably also influences the QoL of pets (dogs), because an owner is less likely to walk or run with their dog in hot weather [18].

The dog–owner relationship may be a source of bias in owner reports of their dog’s symptoms, which complicates the work of veterinarians [12]. There is evidence that the beliefs of the owners of normal-weight dogs are different from those of the owners of obese dogs. Kienzle et al. [10] found that the owners of obese dogs were more likely to believe that exercise and balanced dog nutrition were less important than were the owners of normal-weight dogs. Although factors such as older age, gender, breed, and neutering increase the risk of obesity, the disorder is ultimately caused by inappropriate feeding and exercise given by the owners [19]. Bland et al. [20] concluded that obesity in dogs is affected by the interrelationship between food management, exercise, and social factors such as the belief of owners that obesity is not a health problem. Further, it has been found that the extent to which dogs are overweight is related to the body mass index of their owners [21]. Sandøe et al. [22] states that: “We cannot hope to understand feline and canine obesity without also knowing something about human obesity, the social status of owners, and the relationships that humans actually have with their dogs and cats”. Obesity in pets and humans is thus a One Health issue [22, 23].

Rohlf et al. [6] designed a questionnaire to determine the extent to which owners have perceived control over the feeding and exercise of their pets. The questionnaire is based on the theory of planned behaviour (TPB) [24] which holds that many behaviours, including behaviours towards animals [25], are predicted primarily by intentions, which in turn are determined by behavioural attitudes, subjective norms, and perceived behaviour control. In humans, TPB explains healthy eating and exercise behaviours [26].

How owners feed and exercise their dogs is probably culturally determined, reflecting a composite of subjective beliefs, religion, normative beliefs, and attitudes [16], although there has been relatively little research in this field. In the Netherlands, with its Judeo-Christian tradition [27], food, feeding, and consumptive practices are important constructs of self-identity. In contrast, in Buddhism, food is a component of positive karma, essential to a being’s happiness [28].

Although the assessment of QoL in animals is controversial, it can be measured with well-designed, reliable, and valid questionnaires [12, 29, 30]. Assessment of a dog’s QoL can make it possible to assess the impact of discomfort and disease [31]. Obesity may diminish a dog’s QoL if it causes physical impairments and disabilities.

The aims of this study were to investigate whether BCS and/or culture (the Netherlands and Thailand) affect the owner-reported QoL of dogs and whether the BCS is influenced by owner attitudes regarding feeding and exercise or the owner’s culture.

## Methods

### Data collection and questionnaire

Data were collected at Kasetsart University Veterinary Teaching Hospital (KU-VTH), Thailand, and at veterinary practices and dog grooming parlours in the Netherlands. When the dogs were brought to the KU-VTH or Dutch veterinary practices (such as for vaccination, annual health check-up, spaying/neutering, blood donation, etc.), or to a grooming parlour, their owners were given a questionnaire (see Additional file 1: Appendix) to complete. The owners were informed that this was a study about the quality of life of dogs. The owners were given the questionnaire by the researcher and had to fill in the questionnaire on their own. To prevent that social desirable answers were given, in the introduction letter to the questionnaire it was stated that confidentiality and data protection are promised [32]. The veterinary technician or veterinary student were asked to select dogs that were normal weight (BCS 3), overweight (BCS 4) or obese (BCS 5), but otherwise healthy (Thailand *n =* 200, the Netherlands *n =*155). The veterinary technician or veterinary student were supervised by a veterinarian who was also available for consultation. The veterinarian/veterinary technician/veterinary student have been trained on body condition scoring. In Thailand the dogs that were used for this investigation, came to the Veterinary Teaching Hospital of Kasetsart in Bangkok. In the Netherlands the dogs came from private practices or grooming parlours and the veterinarians or veterinary students who assessed the animals were all educated at the University Clinic for Companion Animal Health in Utrecht, the Netherlands. Veterinarians in both countries were informed about the inclusion criteria, and selected the dogs accordingly. In both countries, pre-selection of dogs was done by trained veterinary technicians or veterinary students, before the veterinarian selected the dogs. To determine the BCS, the BCS chart from Hill's Pet Nutrition (5 scales silhouette system; http://lasvegaspetweightloss.com/hills-metabolic-diet-program/) was used and the BCS was noted at the beginning of the questionnaire. Scores on this scale determined by different operators have been shown to correlate well, although a degree of expertise is required; which makes the scoring instrument less suitable for owners [2, 8].

**Fig. 1:**
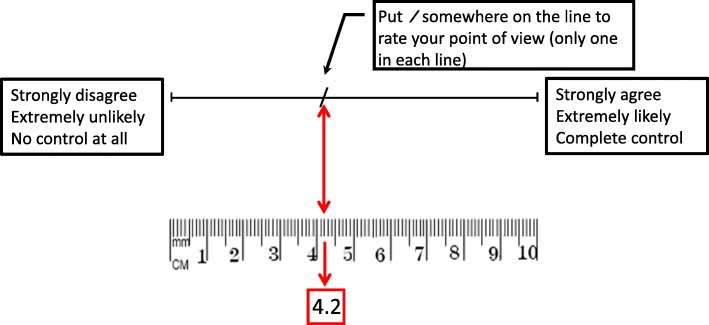
Continuous rating scale used for parts 3 and 4 of the questionnaire (see Additional file 1: Appendix)

The questionnaire contained four sections: one about demographic variables, one about the dog’s food and exercise, one about the dog’s QoL (based on the 91-item questionnaire of Schneider et al. [12]; 39 items selected), and one about owner attitudes (based on the 89-item questionnaire of Rohlf et al. [6]; 42 items selected). The original questionnaire of Schneider et al. [12] has been validated, but that of Rohlf et al. [6] has not. Answers were given on a continuous rating scale (visual analogue scale, Fig. 1). The questionnaire was prepared in English and then translated into Thai and Dutch. Although Chen & Boore [33] advise bilingual translators, the Thai translation was made by five Thai native speakers who were selected from different fields of expertise, such as veterinarians, veterinary technicians, owners graduated in engineering and science, but none of these people were bilingual – they had learned English at school and their study at the university; they were fluent in English. The questionnaire was pre-tested among 11 volunteers in Thailand, as advised by Schellingerhout et al. [34] to increase the validity of the translated questionnaire. It took them about 15 minutes to complete. After adjustment, the questionnaire was distributed among dog owners coming to the KU-VTH. The questionnaire was translated from English into Dutch by the first author together with an English teacher (Dutch native speaker) and three veterinary students. None of these translators were bilingual, but they were fluent in English. The Dutch questionnaire was also pre-tested among 10 dog owners with different educational backgrounds; it took them about 15 minutes to complete. In the pre-testing period, the respondents were asked if they understood the questions. Both the Thai and Dutch questionnaires were in general considered clear so that only minor changes in wording were made. Five questions from part 3 (*‘My dog has trouble going up and/or down stairs’*, *‘My dog has difficulty getting in and/or out cars’*, *‘It is easy to train my dog’*, *‘My dog will perform tricks’*, and *‘My dog responds to verbal correction when he/she misbehaves’*) were left out because they were not specific and could not be used to assess QoL in an overweight and obese population.

### Processing of the questionnaires and z-score calculation

The questionnaires of Rohlf et al. [6] and Scheider et al. [12] used 7-point and 5-point Likert-type scales, respectively. However, Likert-type scales yield ordinal data and thus non-parametric statistical tests should be used, but in general non-parametric tests are less powerful than parametric tests and are also restricted in their application. This implies that parametric compared to non-parametric tests are often inferior. A continuous rating scale makes it possible to use robust parametric statistics and results obtained are less affected by noise. For this reason, the items in the third and fourth parts of the questionnaire were scored on a 10-cm visual analogue scale, going from 0 (‘strongly disagree’, ‘extremely unlikely’, or ‘no control at all’ ) to 10 (‘strongly agree’, ‘extremely likely’, or ‘complete control’) (Fig. 1). Use of a continuous scale made it possible to calculate means and standard deviations (SDs) for the items, so that data could be reduced by creating summary/composite variables.

To obtain comprehensive and integrated summary variables, the data for the questionnaire items were transformed using a z-score methodology. Briefly, for each questionnaire item, z-scores for individual respondents were calculated using the formula below, which indicates how many SD (σ) a rating (X) is above or below the mean (μ) of the pooled data (all the respondents together).

$$ \mathrm{z}=\kern0.5em \left(\mathrm{X}-\upmu \right)/\upsigma $$

The directionality of the z-scores was adjusted so that higher score values reflected higher values for the subscale to which the questionnaire item belonged. The z-scores for questionnaire items were first averaged within a subscale and then across subscales to ensure equal weighting of the subscales comprising the final combined z-score for a specific scale.

### Statistical analyses

All statistical analyses were carried out using an IBM® SPSS® Statistics for Windows (version 22.0) computer program (IBM Corp., Armonk, NY, USA) and paying attention to the assumptions that underlie the various statistical procedures. Two-sided, exact (i.e., for the non-parametric tests) probabilities were estimated throughout. The categorical data from parts 1 and 2 of the questionnaire (see Additional file 1: Appendix) are presented as scores (number of cases) with in parentheses the relative frequency (%). Many factors differ between the Dutch and Thai parts of the study, and in the statistical analyses some of these factors may form part of the factor culture/country whereas others, such as climate, may be confounding environmental factors. Log-linear models were used to analyse categorical data, to detect possible associations between the examined categorical variables and the categorical factors *country* (= Thailand or the Netherlands = culture) and *BCS* ( = 3, 4, or 5).

The continuous data from parts 1 and 2, as well as the integrated z-score data, are expressed as means with standard error of the mean (SEM). The Kolmogorov-Smirnov one-sample test was used (for continuous data) per group and revealed that some continuous variables were not normally distributed. The data for these variables were transformed to a Gaussian distribution, using a suitable mathematical function (logarithmic, logistic, square root or inverse transformation). If this was not possible, the data were rank transformed. Rank transformations are a bridge between parametric and non-parametric statistics [35].

Continuous data were compared with a two-way analysis of variance (ANOVA) with main factors *country* and *BCS*. An assumption for an ANOVA is that the residuals have a normal distribution. As some of the residuals were not normally distributed, the data were mathematically transformed (logarithmic, logistic, square root or inverse transformation). If this was not possible, the continuous data were rank transformed. For all ANOVAs, homoscedasticity was tested with Levene’s test. When necessary, the variances were equalized by logarithmic, logistic, square root or inverse transformation. After transformation, the variances should be similar and the transformed within-group data should be normally distributed. If this was not the case, the data for the continuous variable in question were rank transformed [35]. An ANOVA performed on ranked data is also known as the Scheirer-Ray-Hare extension of the Kruskal-Wallis test [36].

As the dog’s gender and sexual status, age, and duration of ownership may influence scores in parts 3 and 4 (see Additional file 1: Appendix), the integrated z-score data were also tested for significant differences by a four-way analysis of covariance (ANCOVA) with main factors *country*, *BCS*, *dog’s gender*, and *sexual status*. *Age of the dog* and *duration of dog ownership* served as covariates. For the ANCOVAs, homoscedasticity was also tested with Levene’s test.

*Post hoc* comparisons for normally distributed continuous data were performed with the unpaired Student’s *t* test. The Student's *t* tests were performed using pooled (for equal variances) or separate (for unequal variances) variance estimates. The equality of variances was tested with Levene's test. For the unpaired Student's *t* test with separate variance estimates, IBM® SPSS® Statistics uses the Welch-Satterthwaite correction. *Post hoc* comparisons for non-normally distributed continuous data were performed with the Wilcoxon-Mann-Whitney test.

Spearman coefficient of rank correlation (*R*_*S*_) was calculated for: *i)* dog age and ownership; *ii)* dog age and BCS; *iii)* the z-score for immobility and reported total activity (indoor plus outdoor); *iv)* dog age and z-score for general sickness. In addition Pearson’s linear correlation coefficient (*r*) was calculated for logarithmically transformed dog age (*y* = ^10^Log[*x* + 12]) and logarithmically transformed z-score for general sickness (*y* = ^10^Log[*x* + 0.7]. Significance was assessed by a two-tailed test based on the *t* statistic.

In addition, the data from parts 3 (QoL) and 4 (owner attitude) were also analysed by factor analysis, using a principal components solution with orthogonal rotation (varimax) of the factor matrix. This method ensures that the extracted factors are independent of one another and should, therefore, reflect separate processes. The varimax algorithm was chosen, because it attempts to minimize the number of variables that have high loadings (see hereafter) on a factor, which should improve interpretability. Interpretation of a factor analysis is only meaningful if all of the variables are assumed to be scaled at the numeric level (continuous scale of measurement or count based measures), which was the case for the answers to the questions of parts 3 and 4 of the questionnaire (see *Processing of the questionnaires and z-score calculation*). Our objective was data reduction and therefore principal component analysis was used instead of principal axis analysis [37].

Sampling adequacy was measured with the Kaiser-Meyer-Olkin measure (should be greater than ≈0.5). The Bartlett’s test of sphericity was used to test whether the correlation was appropriate for factor analysis. Factor pattern matrices were identified using a fixed number of factors (based on the number of subscales: 9 for QoL data and 11 for owner attitude data; or based on the number of subscales per scale: 2 for the psychological, social and environmental scales, 3 for the physical scale, 5 for the attitude in feeding scale and 6 for the attitude in exercise scale). All extracted factors had an eigenvalue ≥ 1. The factor loading of each question indicated how well that question correlated with the factor; thus a loading of ±1.0 indicates a perfect (positive/negative) correlation, whereas an absolute loading of less than 0.4 would suggest that the question is rather weakly linked to the factor.

The highest (absolute) factor loadings based on total number of subscales (Tables 3 and 4) were compared with those based on number of subscales per scale (Additional file 1: Tables S3 and S4) using a paired Student’s *t* test, since the difference between the two compared sets of (absolute) factor loadings was normally distributed. Schneider et al. [12] conducted a principal axis factoring extraction with varimax rotation. (Absolute) factor loadings for QoL in the present study using a principal axis solution with varimax rotation (results not shown) were also compared (paired Student’s *t* test) to those reported by Schneider et al. [12]; again the difference between the two compared sets of (absolute) factor loadings was normally distributed.

**Fig. 2.**
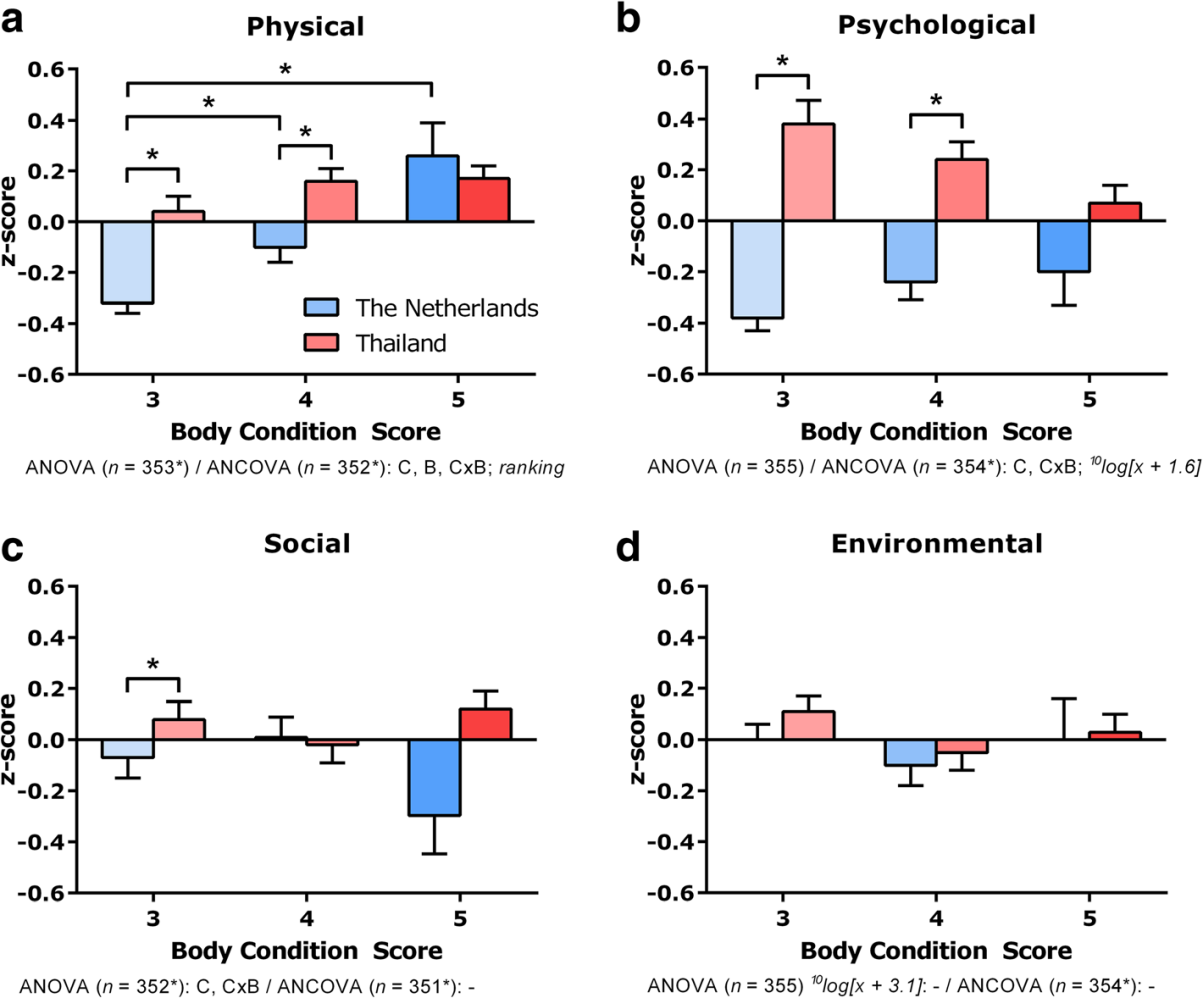
Owner reports of quality of life: scale z-scores. This figure is based on part 3 of the questionnaire (see Additional file 1: Appendix) and the subscale z-scores summarized in Additional file 1: Table S5. Panel **a**, Physical; panel **b**, Psychological; panel **c**, Social; panel **d**, Environmental. The subscale z-scores were averaged, resulting in a scale z-score. Results are presented as means ± SEM. ANOVA = two-way analysis of variance with main factors *country* and *body condition score*. C indicates effect of country; B, effect of body condition score; CxB, interaction; – = no significant C, B and CxB effect. Effects were significant when *P* < 0.05. Scale z-scores that were not normally distributed and/or where the variances were unequal, were first transformed. In the figure the type of transformation is indicated. The data were also tested for significant (*P* < 0.05) differences by an ANCOVA with main factors *country* and *body condition score*, *dog’s gender* and *sexual status*. Covariates were *age of the dog* and *duration of ownership*. *Post hoc* testing was done by unpaired Student’s *t* test (Gaussian distributed data + homoscedasticity), unpaired Student’s *t* test with Welch-Satterthwaite correction (Gaussian distributed data + heteroscedasticity) or Wilcoxon-Mann-Whitney test (non-Gaussian distributed data). * = Significant difference (*P* < 0.005683) in the *post hoc* comparison

**Fig. 3:**
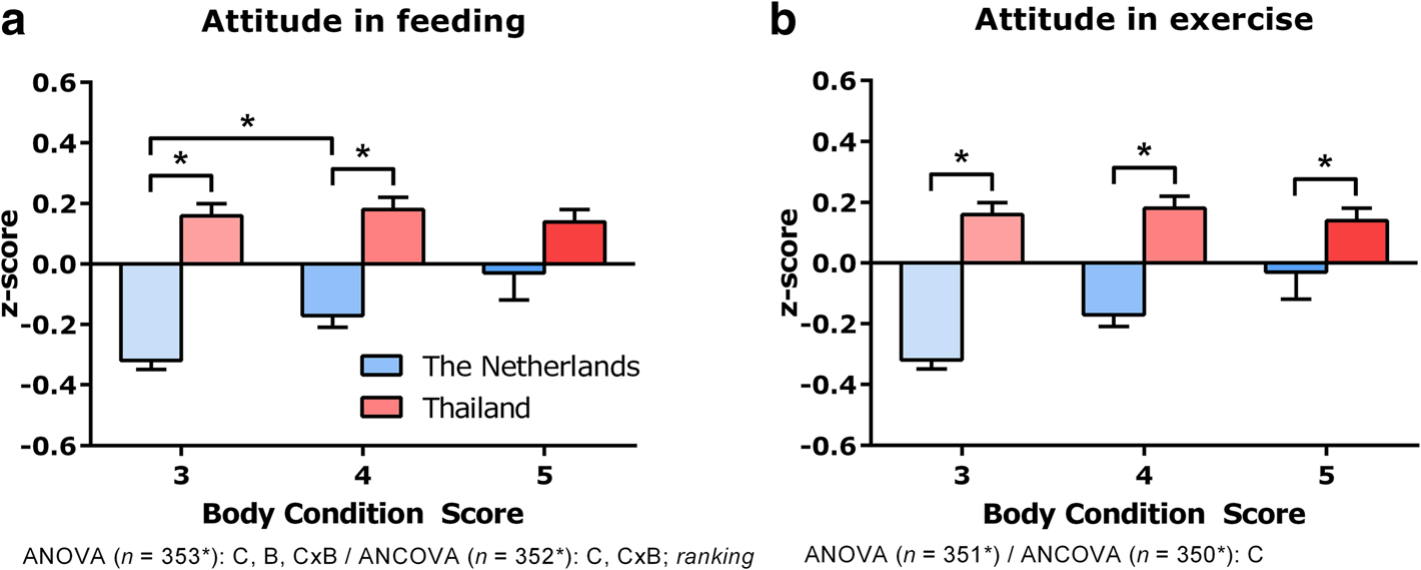
*Owner attitude: scale z-scores.* This figure is based on *part 4* of the questionnaire (see Additional file 1: Appendix) and the subscale z-scores summarized in Additional file 1: Table S6. Panel **a**, Attitude in feeding; panel **b**, Attitude in exercise. The subscale z-scores were averaged, resulting in a scale z-score. Results are presented as means ± SEM. ANOVA = two-way analysis of variance with main factors *country* and *body condition score*. C indicates effect of country; B, effect of body condition score; CxB, interaction; – = no significant C, B and CxB effect. Effects were significant when *P* < 0.05. Scale z-scores that were not normally distributed and/or where the variances were unequal, were first transformed. In the figure the type of transformation is indicated. The data were also tested for significant differences by an ANCOVA with main factors *country* and *body condition score*, *dog’s gender* and *sexual status*. *Post hoc* testing was done by unpaired Student’s *t* test (Gaussian distributed data + homoscedasticity), unpaired Student’s *t* test with Welch-Satterthwaite correction (Gaussian distributed data + heteroscedasticity) or Wilcoxon-Mann-Whitney test (non-Gaussian distributed data). * = Significant difference (*P* < 0.005683) in the *post hoc* comparison

To account for the greater probability of a Type I error due to multiple comparisons, a more stringent criterion should be used for statistical significance (i.e. for the Student's *t* tests and Wilcoxon-Mann-Whitney tests). We approached this problem by calculating a so-called Dunn-Šidák correction (α = 1 - [1 - 0.05]^1/γ^; γ = number of meaningful comparisons; Tables 1 and 2, Additional file 1: Tables S1, S2, S5, S6 and S7 plus Figs. 2 and 3: γ = 9 → α = 0.005683). In all other cases, the probability of a Type I error < 0.05 was taken as the criterion of significance.

**Table 1:** Dog feeding behaviour and sexual status: categorical data^1^

	Body condition score (BCS)						
Country:	The Netherlands			Thailand			
Measure *(number of answers)*/*Category*	BCS 3	BCS 4	BCS 5	BCS 3	BCS 4	BCS 5	Log linear analysis significance^3^
Quantity of food (*n* = 355)^5^	A^4^	B^4^		A	B		
*As it wants*	4(6.0%)^2^	5(8.1%)	2(7.7%)	29(43.9%)	14(20.9%)	26(38.8%)	
*As stated/calculated by veterinarian*	33(49.3%)	29(46.8%)	9(34.6%)	13(19.7%)	14(20.9%)	14(20.9%)	
*As estimated*	30(44.8%)	27(43.5%)	15(57.7%)	24(36.4%)	38(56.7%)	27(40.3%)	
*Do not know*	0(0.0%)	1(1.6%)	0(0.0%)	0(0.0%)	1(1.5%)	0(0.0%)	C,B,M,CxB,MxC
Dog sexual status (*n* = 355)	A			Aa		a	
*Male neutered*	14(20.9%)	15(24.2%)	11(42.3%)	11(16.7%)	13(19.4%)	15(22.4%)	
*Male sexually intact*	20(29.9%)	14(22.6%)	6(23.1%)	33(50.0%)	22(32.8%)	19(28.4%)	
*Female neutered*	20(29.9%)	21(33.9%)	5(19.2%)	5(7.6%)	15(22.4%)	20(29.9%)	
*Female sexually intact*	13(19.4%)	12(19.4%)	4(15.4%)	17(25.8%)	17(25.4%)	13(19.4%)	C,B,M,CxB,MxC

^1^This table is based on *part 1* and *part 2* of the questionnaire (see Additional file 1: Appendix)

^2^Results are presented as scores (number of cases) with in parentheses the relative frequency (%)

^3^Significance (*P* < 0.05) based on log linear analysis with categorical variables/factors *measure* (first column in this table), *country* and *body condition score*. C indicates significant contribution of the factor *country* to the log linear model; B, significant contribution of the factor *body condition score*; CxB, significant contribution of the interaction between the factors *country* and *body condition score*; MxB, significant contribution of the interaction between the variable *measure* and factor *body condition score*; MxC, significant contribution of the interaction between the variable *measure* and factor *country*

^4^Contrast significance (*post hoc* comparisons, *P* < 0.005683). *Post hoc* testing was done by Fischer’s Exact test. Within the same country values with the same superscript lowercase letter were significantly different. Within the same category of body condition score values with the same superscript uppercase letter were significantly different

^5^The number of answers is given in parentheses

**Table 2:** Owner demographics, dog information and activities: continuous data^1^

Measure		Body condition score (BCS)			
(number of answers)^5^	Country	BCS 3	BCS 4	BCS 5	*Transformation*/ANOVA significance^3^
Owner age (years)					
(*n* = 355)	Netherlands	42.0±1.9^**2**^	44.1±1.8	47.8±3.4	*Ranking*
	Thailand	37.6±1.4	38.5±1.6	38.2±1.5	C
Ownership (years)					
(*n* = 354^*^)	Netherlands	5.4±0.5	5.5±0.5	7.3±0.6	*Ranking*
	Thailand	3.9±0.2^a4^	4.7±0.2	5.3±0.2^a^	C,B
Dog age (years)					
(*n* = 355)	Netherlands	5.8±0.5^A^	6.2±0.5	8.2±0.7^B^	*Ranking*
	Thailand	4.2±0.2^aA^	5.1±0.2^b^	5.6±0.2^abB^	C,B,CxB
Total indoor activity (h/week)					
(*n* = 353^*^)	Netherlands	8.9±1.4^aA^	5.3±1.0^B^	3.7±1.7^aC^	*Ranking*
	Thailand	47.9±4.4^A^	41.3±4.4^B^	40.1±5.5^C^	C,B,CxB
Total outdoor activity (h/week)					
(*n* = 353^*^)	Netherlands	19.7±1.7	18.3±1.8^A^	20.8±3.7	*Ranking*
	Thailand	21.1±3.3	13.1±2.1^A^	20.5±3.8	C
Total indoor plus outdoor activity (h/week)					
(*n* = 352^*^)	Netherlands	28.6±2.7^A^	23.6±2.3^B^	24.5±4.1^C^	*Ranking*
	Thailand	69.0±5.7^A^	54.4±4.4^B^	61.6±8.2^C^	C,B

^1^This table is based on *part 1* and *part 2* of the questionnaire (see Additional file 1: Appendix)

^2^Results are presented as means ± SEM

^3^Significance (*P* < 0.05) based on two-way ANOVA with main factors *country* and *body condition score*. C indicates effect of *country*; B, effect of *body condition score*; CxB, interaction. Measures that are not normally distributed and/or where the variances were unequal were first transformed. In this column the type of transformation is also given

^4^Contrast significance (*post hoc* comparisons, *P* < 0.005683). *Post hoc* testing was done by unpaired Student’s *t* test (Gaussian distributed data + homoscedasticity), unpaired Student’s *t* test with Welch-Satterthwaite correction (Gaussian distributed data + heteroscedasticity) or Wilcoxon-Mann-Whitney test (non-Gaussian distributed data). Within the same row (country) values with the same superscript lowercase letter were significantly different. Within the same column (body condition score) values with the same superscript uppercase letter were significantly different

^5^The number of answers is given in parentheses

^*^Indicates missing answers

## Results

### General

In total, 200 Thai dog owners and 155 Dutch dog owners completed the questionnaire. There were few missing answers (this information can be found in Tables 1 to 4, Additional file 1: Tables S1 to S6 plus Figs. 2 and 3), indicating that the owners experienced no difficulty in understanding/completing the questionnaire. Information about owner demographics, dog-feeding behaviour, and dog information can be found in Table 1 and Additional file 1: Table S1. Only sixteen respondents (≈ 4.5%) were not totally or mostly (‘child of owner’ or ‘caretaker’) in charge of dog management (Additional file 1: Table S1).

Unfortunately, in the Netherlands it was very difficult to find dogs with a BCS of 5 that did not have another diseases besides obesity or overweight. There was no difference between BCS groups in the way the food was administered (log linear analysis *interaction between ‘quantity of food’ and ‘BCS’*: partial Chi-square = 10.621, *df* = 6, *P* = 0.100809), although Thai dogs were more often fed *ad libitum* than were Dutch dogs (log linear analysis *interaction between ‘quantity of food’ and ‘country’*: partial Chi-square = 47.842, *df* = 3, *P* < 0.0000005*) (Table 1). Male, sexually intact Thai dogs had a BCS of 3 more often than did neutered Thai male dogs, and sterilized female Thai dogs more often had a BCS of 5 than did non-sterilized female Thai dogs (*post hoc* analysis, Fisher’s Exact test: *P* = 0.002622*), but these differences were not seen in the Dutch dogs (*post hoc* analysis, Fisher’s Exact test: *P* = 0.240207) (Table 1).

Among the Thai dogs, there was a significant difference in years of ownership between the dogs in BCS groups 3 and 5 (*post hoc* analysis, unpaired Student’s *t* test, *t*_*131*_ = -4.032, *P* = 0.000093*). As years of ownership was significantly (*P* < 0.0000005*) correlated with dog age in Thailand (Additional file 1: Figure S1, panel A: *R*_*S*_ = 0.898), the older the dog the higher its BCS score tended to be (see Table 2 and Additional file 1: Figure S2: *R*_*S*_ = 0.302, *P* = 0.000014*). A similar trend was seen among Dutch dogs (Additional file 1: Figure S1, panel B: *R*_*S*_ = 0.906, *P* < 0.0000005*; Additional file 1: Figure S2: *R*_*S*_ = 0.193, *P* = 0.016343*). When looking at the effect of BCS on exercise, we distinguished between indoor (i.e. in house) and outdoor exercise. Although we thought that dogs in Thailand in general would have less outdoor exercise than dogs in the Netherlands, this was only the case for dogs in BCS group 4, based on the total outdoor activity score (Table 2; *post hoc* analysis, Wilcoxon-Mann-Whitney test: *U* = 1333.5, *W* = 3611.5, *Z* = -3.519, *P* = 0.000375*). However, the total indoor activity of Thai dogs was significantly higher than that of Dutch dogs (Table 2; ANOVA *country*: rank-transformed, *F*_*1,347*_ = 11.935, *P* = 0.000619*), so that the Thai dogs had more exercise overall (Table 2; ANOVA *country*: rank-transformed, *F*_*1,346*_ = 92.736, *P* < 0.0000005*). In *post hoc* comparisons no significant differences (unpaired Student’s *t* test or Wilcoxon-Mann-Whitney test: *P* ≥ 0.005683) were found in overall exercise between the three BCS groups (Table 2). In the Dutch cohort, dogs in the BCS 5 group had significantly less indoor exercise than dogs in the BCS 3 group (*post hoc* analysis, Wilcoxon-Mann-Whitney test: *U* = 481.0, *W* = 832.0, *Z* = -3.388, *P* = 0.000559*), but this difference was not seen in the Thai cohort (*post hoc* analysis, Wilcoxon-Mann-Whitney test: *U* = 1666.5, *W* = 3810.0, *Z* = -2.209, *P* = 0.026942) (Table 2). There were some differences between BCS groups in the type of exercise; these results are presented in Additional file 1: Table S2.

### Orthogonal factor loadings

Principal component factor analysis was used to analyse the owner-reported QoL data (Table 3). Nine factors from the 34 questions accounted for 55.0% of the total variance, with eigenvalues > 1.17. The psychological subscale ‘Anxiety when the owner leaves’ and the environmental subscale ‘Basic needs’ had somewhat low reliability and hence interpretations require some caution. Additional file 1: Table S3 summarizes the results of the principal component factor analyses for owner-reported QoL data, but now per scale, which – as would be expected – clearly improved the reliability of the subscales ‘Anxiety when the owner leaves’ and ‘Basic needs’. However, there was no significant difference in the magnitude of the highest (absolute) factor loading (per question) between data presented in Table 3 and Additional file 1: Table S3 (paired Student’s *t* test, *t*_*33*_ = -1.221, *P* = 0.230780).

**Table 3:** Orthogonal factor loadings for owner-reported quality of life^1^

Scale/*Subscale*/Question number		Orthogonal factors								
		IM	GS	AL/GA	DF/BN	IM/AL	GS	EI	SO/BN	BN/SA
	Item	Fac1	Fac2	Fac3	Fac4	Fac5	Fac6	Fac7	Fac8	Fac9
Eigenvalue		4.86	3.10	2.24	1.74	1.52	1.44	1.35	1.27	1.18
% of the total variance		14.3	9.1	6.6	5.1	4.5	4.2	4.0	3.7	3.5
Physical										
*General sickness (GS)*										
Q1:	*My dog acts sick.*	0.195	**0.539**	0.071	0.084	-0.043	0.158	0.322	-0.081	0.123
Q2:	*My dog does not like being touched.*	0.095	**0.582**	0.021	-0.122	-0.103	0.153	-0.095	0.069	0.181
Q3:	*My dog has difficulty eliminating or eliminates more frequently than usual*.	0.192	**0.661**	0.160	-0.094	0.043	0.018	0.051	0.115	0.022
Q4:	*My dog has difficulty sleeping.*	0.070	**0.671**	0.217	-0.057	0.057	-0.117	0.075	0.030	-0.225
Q5:	*My dog vomits more than he/she used to.*	-0.107	**0.569**	-0.084	-0.021	0.066	0.202	0.135	-0.181	0.023
Q6:	*My dog has difficulty breathing.*	0.395	0.396	-0.058	0.230	0.083	0.182	0.189	-0.235	-0.171
Q7:	*My dog’s temperament has changed.*	0.219	**0.504**	0.120	0.067	-0.016	0.455	-0.077	-0.088	-0.206
Q8:	*My dog has become aggressive to dogs or people who were accepted before.*	-0.024	0.107	0.202	-0.042	0.125	**0.774**	0.033	0.047	-0.062
Q9:	*My dog gets lost in places that should be familiar.*	0.169	0.214	0.005	-0.034	0.172	**0.433**	0.060	-0.092	-0.034
Q10:	*My dog shows less desire to interact with other dogs or people than usual.*	0.263	0.071	0.145	-0.132	0.062	**0.621**	0.190	-0.001	-0.048
*Immobility (IM)*										
Q11:	*My dog has a lot of energy.*	-0.073	-0.100	0.144	-0.084	**-0.692**	-0.168	-0.079	0.036	-0.148
Q12:	*My dog rarely gets excited anymore.*	**0.568**	0.051	0.042	-0.148	0.028	0.166	0.003	0.319	0.120
Q13:	*My dog has difficulty getting up after lying down.*	**0.832**	0.102	-0.084	-0.059	0.068	0.031	0.012	-0.067	0.002
Q14:	*My dog has difficulty walking.*	**0.827**	0.164	-0.036	-0.055	0.013	-0.008	0.073	-0.036	-0.017
Q15:	*My dog plays less often.*	**0.727**	0.069	0.003	0.026	-0.128	0.155	0.021	-0.147	-0.066
Q16:	*My dog’s overall mobility is good (reverse score).*	0.001	0.031	0.012	-0.154	**-0.691**	-0.123	-0.256	0.196	-0.014
*External irritation (EI)*										
Q17:	*My dog chews or scratches certain areas of skin until they become red and irritated.*	0.028	0.045	0.045	-0.010	0.135	0.093	**0.793**	-0.025	0.015
Q18:	*My dog has patches of fur missing.*	0.100	0.157	0.226	-0.020	0.080	0.028	**0.708**	0.033	-0.056
Psychological										
*Anxiety when owner leaves (AL)*										
Q19:	*My dog chews on things that he/she has been taught not to do.*	-0.008	-0.129	0.356	-0.075	**0.594**	-0.002	0.023	0.260	-0.002
Q20:	*My dog whines when I leave.*	-0.043	0.171	**0.419**	0.057	0.271	0.119	-0.003	-0.165	-0.045
Q21:	*My dog makes a mess when I’m away from home.*	-0.097	0.028	**0.474**	-0.020	**0.615**	-0.024	-0.049	-0.004	-0.006
*General anxiety (GA)*										
Q22:	*My dog is startled easily.*	-0.106	0.176	**0.547**	0.037	0.106	0.123	0.361	-0.154	0.111
Q23:	*My dog cowers when it meets a new person or dog.*	-0.041	0.081	**0.718**	0.014	-0.092	0.294	0.044	-0.123	-0.011
Q24:	*When my dog is in a new place, it puts its tail between its legs.*	0.045	0.045	**0.759**	0.027	0.038	-0.031	0.130	-0.112	0.002
Social										
*Dog focused (DF)*										
Q25:	*I play with my dog when it is ready to play.*	0.145	0.036	0.076	**0.446**	0.080	-0.119	-0.062	0.187	0.042
Q26:	*I pet my dog often.*	-0.022	-0.078	0.014	**0.757**	0.014	-0.037	-0.048	0.032	-0.001
Q27:	*I groom my dog often.*	-0.037	-0.049	-0.043	**0.670**	-0.018	0.046	0.142	0.050	0.091
Q28:	*I often spend my free time with my dog.*	-0.255	0.011	0.024	**0.686**	0.135	-0.078	-0.040	0.073	-0.085
*Sociability (SO)*										
Q29:	*My dog has the opportunity to play with other dogs.*	-0.067	-0.021	-0.146	0.177	0.098	-0.179	-0.029	**0.662**	-0.025
Q30:	*My dog shares his/her toys with other animals.*	-0.049	-0.038	-0.194	0.208	-0.096	-0.024	0.074	**0.718**	-0.072
Environmental										
*Basic needs (BN)*										
Q31:	*My dog has fresh water available throughout the day.*	-0.056	-0.275	0.087	**0.464**	-0.140	0.079	-0.055	0.021	**0.511**
Q32:	*My dog goes outside when he/she needs to.*	-0.038	0.021	-0.109	0.003	-0.121	0.317	-0.098	**0.465**	0.093
*Sleeping area (SA)*										
Q33:	*My dog’s sleeping area is her/her own.*	0.068	0.013	-0.036	-0.055	-0.017	-0.249	-0.113	0.009	**0.729**
Q34:	*My dog has his/her own place to sleep (e.g. bed of the owner).*	-0.085	0.106	0.031	0.095	0.318	0.055	0.169	-0.033	**0.675**

^1^The data from the owner reports of quality of life (*n* = 350) were subject to factor analysis. The Kaiser-Meyer-Olkin measure is 0.741, indicating a high sampling adequacy for the factor analysis. Bartlett’s test of sphericity indicates that the factor model is appropriate (Chi-square = 2934.523, *df* = 561, *P* < 0.0005). Factor

loadings > 0.4 are considered to be high and are indicated in bold. The nine factors account for 55.0% of the total variance

*GS* general sickness, *IM* immobility, *EI* external irritation, *AL* anxiety when owner leaves, *GA* general anxiety, *DF* dog focused, *SO* sociability, *BN* basic needs, *SA* sleep area

The orthogonal factor loadings for owner attitude are shown in Table 4. The 11 factors extracted from the 42 questions accounted for 72.0% of the total variance. The attitude in exercise-subscale ‘Owner centred’ had somewhat low reliability and hence interpretation requires some caution. Additional file 1: Table S4 shows the results of the principal component factor analyses per scale for owner attitude, which improved the reliability of the subscale ‘Owner centred’. On average, the (absolute) factor loadings of data in Table 4 were 1.8 times (and significantly) higher than those of data in Additional file 1: Table S4 (paired Student’s *t* test, *t*_*41*_ = -2.269, *P* = 0.028580*).

**Table 4:** Orthogonal factor loadings for owner attitude^a^

		Orthogonal factors										
		FCB										
Scale/*Subscale*/Question number		OC			OE		AK		FP			OE
		ECB	OC	EB	DB	AK	VE	VE	OC	DC	LK	DB
	Item	Fac1	Fac2	Fac3	Fac4	Fac5	Fac6	Fac7	Fac8	Fac9	Fac10	Fac11
Eigenvalue		11.22	3.73	2.63	2.20	2.10	1.95	1.52	1.44	1.31	1.17	1.00
% of the total variance		26.7	8.9	6.3	5.2	5.0	4.6	3.6	3.4	3.1	2.8	2.4
Attitude in feeding												
*Ambivalence about knowledge (AK)*												
Q35:	*I don’t know how much food to feed my dog.*	-0.144	0.127	0.139	0.227	**0.691**	0.191	-0.107	0.009	0.039	0.223	0.027
Q36:	*I don’t know what type of food to feed my dog.*	-0.124	0.101	0.027	0.150	**0.778**	0.181	-0.101	0.077	0.045	0.174	-0.028
Q37:	*I don’t know how many times a day I should feed my dog.*	-0.101	0.064	-0.018	0.101	**0.807**	0.026	-0.037	0.116	0.050	0.190	0.082
Q38:	*It’s important that I feed my dog the appropriate type of food.*	0.210	-0.078	-0.040	-0.077	-0.125	**-0.839**	-0.082	-0.112	-0.090	-0.114	-0.092
Q39:	*It’s important that I feed my dog the appropriate number of times a day.*	0.177	-0.138	-0.038	-0.161	-0.086	**-0.840**	-0.051	-0.139	-0.097	-0.105	-0.056
*Feed to please (FP)*												
Q40:	*It’s important that I feed my dog whenever he/she likes.*	-0.049	0.005	0.057	0.107	0.026	0.158	-0.070	**0.778**	0.088	0.140	0.094
Q41:	*It’s important that I feed my dog whatever he/she likes.*	-0.069	0.068	0.081	0.318	0.160	0.021	-0.074	**0.695**	0.242	-0.017	-0.063
Q42:	*It’s important that I feed my dog as much as he/she wants.*	-0.116	0.066	0.003	0.227	0.043	0.304	-0.001	**0.690**	0.240	0.066	-0.038
*Owner-centred / External barrier (OE)*												
Q43:	*My dog is overfed because he/she always wants food.*	-0.220	0.176	0.005	**0.546**	0.076	-0.004	-0.115	0.265	0.004	0.061	0.255
Q44:	*My dog isn’t given the appropriate type of food because others feed the dog.*	-0.197	0.129	0.141	0.114	-0.014	0.108	-0.001	0.060	0.038	0.023	**0.800**
Q45:	*I feed my dog inappropriate types of food because he/she likes that kind of food.*	-0.199	0.110	0.161	**0.655**	0.100	0.197	-0.054	-0.024	0.147	0.144	0.165
Q46:	*My dog isn’t fed the appropriate number of times per day because others feed him/her.*	-0.040	0.076	0.334	0.240	0.048	0.079	-0.085	-0.054	0.101	0.086	**0.690**
*Dog-centred barriers (DB)*												
Q47:	*I feed my dog inappropriate food because I like to spoil him/her.*	-0.091	0.224	0.053	**0.752**	0.198	0.101	-0.005	0.142	0.008	0.067	0.079
Q48:	*My dog is overfed because I indulge him/her.*	-0.229	0.175	0.050	**0.718**	0.182	0.040	-0.058	0.275	-0.014	0.147	0.041
Q49:	*I feed my dog inappropriate food because appropriate food is too expensive.*	-0.018	0.270	-0.069	0.308	0.243	-0.133	-0.055	0.276	-0.028	0.023	0.371
*Control belief (FCB)*												
Q50:	*Overall, how much control do you feel you have over the amount you feed your dog.*	**0.615**	0.004	-0.098	-0.307	-0.206	-0.146	0.186	-0.181	-0.078	-0.040	-0.163
Q51:	*Overall, how much control do you feel you have over the type of food you feed your dog.*	**0.553**	0.023	-0.118	-0.191	-0.244	-0.132	0.261	-0.173	-0.056	-0.106	-0.275
Q52:	*Overall, how much control do you feel you have over the number of times you feed your dog during the day.*	**0.537**	0.123	-0.084	-0.268	-0.281	-0.058	0.270	-0.096	0.002	-0.096	-0.292
Attitude in exercise												
*Value exercise (VE)*												
Q53:	*It’s important that I exercise my dog the appropriate number of times a week.*	0.166	-0.177	-0.002	-0.021	-0.028	0.036	**0.822**	0.045	0.076	-0.072	0.003
Q54:	*It’s important that I give my dog the appropriate type of exercise.*	0.102	-0.131	-0.066	-0.090	-0.066	-0.010	**0.838**	0.012	0.029	-0.143	-0.021
Q55:	*It’s important to me that my dog is fit.*	-0.044	-0.068	0.040	0.024	-0.116	0.057	**0.729**	-0.120	0.136	0.058	-0.151
Q56:	*My dog doesn’t need exercise.*	-0.200	-0.026	0.020	0.020	0.165	**0.747**	0.067	0.167	0.120	0.190	0.018
Q57:	*It is important that I exercise my dog for the appropriate length of time.*	-0.045	-0.082	-0.069	-0.154	-0.001	0.218	**0.485**	-0.064	0.190	0.268	0.151
*Lack of knowledge (LK)*												
Q58:	*I don’t know how often I should exercise my dog.*	-0.235	0.163	0.103	0.136	0.348	0.184	-0.035	0.180	0.067	**0.697**	0.053
Q59:	*I don’t know the appropriate length of time my dog should be exercised.*	-0.270	0.175	0.106	0.158	0.288	0.174	-0.054	0.129	0.056	**0.762**	0.067
Q60:	*I don’t know what type of exercise to give my dog*.	-0.197	0.187	0.088	0.182	0.304	0.222	0.000	0.073	0.034	**0.760**	0.050
*Dog centred (DC)*												
Q61:	*It’s important that I exercise my dog as frequently as he/she wants.*	-0.125	0.011	0.009	0.050	-0.046	0.215	0.130	0.188	**0.768**	0.096	0.040
Q62:	*It’s important that I exercise my dog for as long as he/she wants.*	-0.054	0.039	-0.038	-0.015	0.082	-0.014	0.079	0.105	**0.880**	0.082	0.010
Q63:	*It’s important that I give my dog the type of exercise that he/she likes.*	-0.081	-0.048	0.058	0.067	0.063	0.099	0.117	0.068	**0.793**	-0.051	0.050
*Owner centred (OC)*												
Q64:	*I don’t exercise my dog frequently enough because I don’t like to.*	-0.121	**0.834**	0.043	0.139	0.155	0.057	-0.111	0.000	-0.014	0.064	0.075
Q65:	*I don’t exercise my dog for long enough because I don’t like to.*	-0.107	**0.848**	0.098	0.154	0.174	0.065	-0.104	0.031	0.033	0.043	0.054
Q66:	*I don’t give my dog the appropriate type of exercise because I don’t like to.*	-0.161	**0.816**	0.099	0.176	-0.008	0.028	-0.134	0.041	0.009	0.119	0.013
Q67:	*I don’t give my dog the appropriate kind of exercise because he/she doesn’t like that type of exercise.*	-0.075	**0.600**	0.151	0.311	-0.103	0.061	-0.217	0.047	0.047	0.176	0.046
Q68:	*I don’t exercise my dog as frequently as I should because he/she is badly behaved.*	-0.105	**0.412**	0.256	-0.147	0.245	0.030	0.006	0.237	-0.139	-0.288	0.219
Q69:	*I don’t exercise my dog as frequently as I should because I don’t have time.*	**-0.460**	**0.509**	0.111	-0.109	0.030	0.027	0.008	0.238	-0.051	0.202	0.173
Q70:	*I don’t give my dog the appropriate type of exercise because I don’t have access to appropriate areas.*	**-0.428**	0.299	0.015	-0.148	0.126	0.097	0.100	**0.477**	-0.113	0.141	0.209
*External barrier (EB)*												
Q71:	*My dog isn’t exercised frequently enough because other people exercise it.*	-0.126	0.123	**0.918**	0.034	0.027	0.044	0.001	0.042	-0.021	0.042	0.051
Q72:	*My dog isn’t given the appropriate type of exercise because other people exercise it.*	-0.095	0.094	**0.911**	0.072	0.055	0.009	-0.041	0.032	0.045	0.072	0.166
Q73:	*My dog isn’t exercised long enough because other people exercise it.*	-0.098	0.127	**0.901**	0.092	0.033	0.028	-0.028	0.048	0.009	0.058	0.117
*Control belief (ECB)*												
Q74:	*Overall, how much control do you feel you have over the type of exercise you give your dog.*	**0.820**	-0.203	-0.075	-0.135	-0.033	-0.186	0.016	0.048	-0.088	-0.121	0.014
Q75:	*Overall, how much control do you feel you have over how frequently you exercise your dog.*	**0.852**	-0.236	-0.085	-0.134	-0.038	-0.156	-0.028	-0.058	-0.118	-0.114	-0.023
Q76:	*Overall, how much control do you feel you have over the length of time you exercise your dog?*	**0.864**	-0.190	-0.090	-0.091	-0.055	-0.149	-0.005	-0.046	-0.108	-0.141	0.017

^a^The data from the owner attitude (*n* = 349) were subject to factor analysis. The Kaiser-Meyer-Olkin measure is 0.872, indicating a high sampling adequacy for the factor analysis. Bartlett’s test of sphericity indicates that the factor model is appropriate (*P* < 0.0005). Factor Loadings > 0.4 are considered to be high and are indicated in bold. The eleven factors account for 72.0% of the total variance

*AK* ambivalence about knowledge, *FP* feed to please, *OE* owner-centred / external barrier, *DB* dog-centred barriers, *FCB* control belief (part of: attitude of feeding), *VE* value exercise, *LK* lack of knowledge, *DC* dog centred, *OC* owner centred, *EB* external barrier, *ECB* control belief (part of: attitude in exercise).

### Quality of life

Because we included only healthy dogs, we did not expect to find differences in QoL scale ‘Physical’ between the BCS groups (Additional file 1: Table S5). However, among the Dutch dogs, BCS was positively associated with ‘General sickness’, indicating that according to Dutch owners overweight and obesity influenced the health status of their dogs (ANOVA, logarithmically transformed: *country*, *F*_*1,348*_ = 8.508, *P* = 0.003765*; *body condition score*, *F*_*2,348*_ = 10.266, *P* = 0.000047*; *interaction, F*_*2,348*_ = 3.136, *P* = 0.044669*). The Dutch dogs with a BCS of 3 had lower scores for ‘General sickness’ than their Thai counterparts (*post hoc* analysis, Wilcoxon-Mann-Whitney test: *U* = 1346.5, *W* = 3624.5, *Z* = -3.892, *P* = 0.000079*). Dog immobility increased with increasing BCS (ANOVA *body condition score*: rank-transformed, *F*_*1,348*_ = 21.098, *P* < 0.0000005*). This effect was more pronounced in the Dutch dogs (ANOVA *interaction*: rank-transformed, *F*_*2,348*_ = 11.432, *P* = 0.000016*): Thai dogs with a BCS of 5 were less immobile than Dutch dogs with the same BCS (*post hoc* analysis, unpaired Student’s *t* test with Welch-Satterthwaite correction, *t*_*28.484*_ = -3.115, *P* = 0.004168*), whereas the opposite was true for dogs with a BCS of 3 (*post hoc* analysis, Wilcoxon-Mann-Whitney test: *U* = 1148.5, *W* = 3426.5, *Z* = -4.782, *P* < 0.0000005*).

Two aspects of anxiety were included in the scale ‘Psychological’, ‘Anxiety when owner leaves’ and ‘General anxiety’ (Additional file 1: Table S5). The Thai dogs were more anxious when their owner left home than were the Dutch dogs (ANOVA *country*: rank-transformed, *F*_*1,349*_ = 12.859, *P* = 0.000384*), but this difference was only significant for dogs with a BCS of 3 (*post hoc* analysis, Wilcoxon-Mann-Whitney test: *U* = 955.5, *W* = 3233.5, *Z* = -5.655, *P* < 0.0000005*) or a BCS of 4 (*post hoc* analysis, Wilcoxon-Mann-Whitney test: *U* = 1201.5, *W* = 3154.5, *Z* = -4.131, *P* = 0.000026*). In the Netherlands, the group mean for ‘Anxiety when owner leaves’ increased with increasing BCS, whereas it decreased in Thailand, suggesting that there was an interaction effect of country and BCS (ANOVA *interaction*: rank-transformed, *F*_*2,349*_ = 8.288, *P* = 0.000304*). On the social and environmental scales (Additional file 1: Table S5), only the factor ‘country’ influenced QoL (‘Dog focused’, ANOVA *country*: non-transformed, *F*_*1,348*_ = 11.644, *P* = 0.000720*; ‘Sociability’, ANOVA *country*: non-transformed, *F*_*1,347*_ = 0.636, *P* = 0.425725; ‘Basic needs’, ANOVA *country*: rank-transformed, *F*_*1,349*_ = 8.378, *P* = 0.004036*; ‘Sleeping area’, ANOVA *country*: non-transformed, *F*_*1,349*_ = 12.975, *P* = 0.000361*).

The data were also tested for significant differences using an ANCOVA with main factors *country*, *BCS*, *dog’s gender,* and *sexual status*; covariates were *dog age* and *duration of ownership*. *Country*, and an interaction between *country* and *BCS*, influenced ‘General anxiety’ (ANCOVA, rank-transformed: *country*, *F*_*1,328*_ = 43.0565, *P* < 0.0000005*; *interaction*, *F*_*2,328*_ = 3.183, *P* = 0.042737*) and ‘Sleeping area’ (ANCOVA, rank-transformed: *country*, *F*_*1,328*_ = 6.050, *P* = 0.014423*; *interaction*, *F*_*2,328*_ = 9.437, *P* = 0.000104*). There was now a main effect of BCS on ‘Sleeping area’ (ANCOVA, rank-transformed: *body condition score*, *F*_*2,328*_ = 4.395, *P* = 0.013068*) (Additional file 1: Table S5).

Combination of the QoL z-scores identified four scales, namely, Physical, Psychological, Social, and Environmental (Fig. 2). In the Dutch cohort, physical scale scores differed by BCS group, with the higher the BCS, the higher the physical scale score (*post hoc* analyses, unpaired Student’s *t* test: BCS 3 *versus* BCS 4, Welch-Satterthwaite correction, *t*_*108.616*_ = -3.721, *P* = 0.000316*; BCS 3 *versus* BCS 5, Welch-Satterthwaite correction, *t*_*28.457*_ = -4.837, *P* = 0.000042*; BCS 4 *versus* BCS 5, no correction, *t*_*84*_ = -2.812, *P* = 0.006124).

### Owner attitude

Country, BCS, and an interaction on all the subscales significantly (ANOVA, rank-transformed, *P* < 0.05*) influenced ‘attitude in feeding’ (Additional file 1: Table S6). The Dutch respondents rated their control of feeding higher than did the Thai respondents, with the owners of dogs with a BCS of 3 reporting the highest control over feeding (ANOVA rank-transformed: *country*, *F*_*1,349*_ = 12.985, *P* = 0.000360*; *body condition score*, *F*_*2,349*_ = 7.830, *P* = 0.000471*; *interaction*, *F*_*2,349*_ = 8.369, *P* = 0.000282*). The Thai owners fed their dogs to please more often than did the Dutch owners (ANOVA *country*: rank-transformed, *F*_*1,349*_ = 12.865, *P* = 0.000383*). This difference was significant for BCS 3 (*post hoc* analysis, Wilcoxon-Mann-Whitney test: *U* = 690.0, *W* = 2968.0, *Z* = -6.857, *P* < 0.0000005*) and BCS 4 (*post hoc* analysis, unpaired Student’s *t* test, no correction, *t*_*127*_ = -4.028, *P* = 0.000096*) dogs.

Only in the Netherlands did the subscales ‘value of exercise’ (*post hoc* analysis, BCS 3 *versus* BCS 5, Wilcoxon-Mann-Whitney test: *U* = 473.0, *W* = 824.0, *Z* = -3.418, *P* = 0.000494*), ‘lack of knowledge about exercise’ (*post hoc* analysis, BCS 3 *versus* BCS 5, Wilcoxon-Mann-Whitney test: *U* = 504.5, *W* = 2782.5, *Z* = -3.156, *P* = 0.001356*), and ‘the fact that owners were more or less self-centred’ (*post hoc* analysis, BCS 3 *versus* BCS 5, Wilcoxon-Mann-Whitney test: *U* = 411.5, *W* = 2689.5, *Z* = -3.940, *P* = 0.000049*) significantly affect BCS (Additional file 1: Table S6). As would be expected, in the Netherlands groups mean scores for ‘value of exercise’ decreased with increasing BCS of the dogs.

Z-scores for the ‘attitude’ subscales were combined to yield two scales, i.e. ‘Attitude in feeding’ and ‘Attitude in exercise’ (Fig. 3). ‘Attitude in feeding’ was significantly different between the Dutch owners of dogs with a BCS of 3 and 4 (*post hoc* analysis, Wilcoxon-Mann-Whitney test: *U* = 1261.5, *W* = 3472.5, *Z* = -3.627, *P* = 0.000240*), and there was a significant between-countries difference in ‘attitude in exercise’ (ANOVA *country*: non-transformed, *F*_*1,345*_ = 32.690, *P* < 0.0000005*). The association between BCS and ‘attitude in feeding’ disappeared on ANCOVA (ANOVA rank-transformed: *body condition score*, *F*_*2,347*_ = 4.894, *P* = 0.008018*; ANCOVA rank-transformed: *body condition score*, *F*_*2,326*_ = 1.611, *P* = 0.201229).

## Discussion

### General

Both the Dutch and Thai questionnaires were designed to be completed by owners while they were waiting for the veterinarian or dog groomer, and most owners were willing to do so. In the Netherlands, it was very difficult to find dogs that were ‘healthy’ despite being overweight or obese (BCS 4/5) and we recruited only 26 dogs with a BCS of 5. Moreover, the Dutch dog owners were less homogeneous than the Thai owners, possibly because the Thai owners were recruited at one site (KU-VTH), whereas in the Netherlands owners were recruited at various veterinary practices or dog grooming parlours in the province of Utrecht.

There were a number of potential confounders, such as differences in climate. In the Netherlands there is a temperate maritime climate with cool summers and moderate winters. Since the country is small there is little variation in climate from region to region. There are 4.5 million inhabitants in the province of Utrecht, whereas there are more than 10 million inhabitants in the metropolis of Bangkok, where the climate is tropical, with high temperature and high humidity. These climatic factors certainly influence the way dogs are kept, how often they are exercised outdoors, etc. For instance, obese dogs may be less tolerant of heat, which may affect the QoL of dogs living in warmer climates [38]. Homes with housekeepers or maids are much more common in Thailand than in the Netherlands. As these people also take care of the dogs, it is possible that the owners do not have all the information about their dog. These confounding factors probably influenced the results and were not taken into account. The reader should bear this in mind.

Body condition was scored with the BCS card of Hill’s Pet Nutrition. Witzel et al. [39] found that morphometric measurements and the body fat index to be more accurate than the 5-point BCS method, but recognized that their approach needed to be validated in other dogs, such as normal and underweight dogs. There are other scoring systems, such as the 7-point SHAPE algorithm. This scoring system has been validated and owners are able to use it without prior training [40]. In the Netherlands, veterinarians work with the 5-point BCS, and the veterinarian who used the BCS in Thailand had been trained to use it in the Netherlands. In this way, we hoped to obtain a good inter-observer reliability. The SHAPE algorithm could be used in future studies.

In Thailand, dog sexual status significantly affected the BCS: intact dogs typically had a BCS of 3 whereas spayed and neutered dogs typically had a BCS of 5 (Table 1). There is strong evidence that dogs gain weight after sterilization ([41] and references cited therein), possibly because they are less active (but with the same energy intake) or they eat more [42]. Moreover, BCS increased with dog age, which was correlated with the duration of dog ownership: the older the dog, the longer it was owned, and the higher the BCS (Table 1). This is not surprising since overweight and obesity is considered a disorder of the middle-aged dogs [41].

### Limitations of translating a questionnaire in a cross-cultural setting

Questionnaires are widely used in veterinary research [43] and generally provide a low-cost method to obtain information about a range of factors. With careful design, assessment and administration, questionnaires can collect accurate data. In cross-cultural settings with respondents that speak different languages – like this study – questionnaire design is complicated by the added step of translation. One of the major problems with cross-cultural research is to ensure that the questions are comparable across cultural groups and that interpretations are not affected by cultural bias. For example wording of questions might have to be changed to ensure comparability of meaning, especially as some words and concepts in one language will have no equivalent in another language. Guidelines exist for designing questionnaires in a cross-cultural setting (see: http://ccsg.isr.umich.edu/index.php/chapters/questionnaire-design-chapter). Regarding translation equivalence there is no gold standard, but variations of Brislin’s classic and iterative back-translation model are most commonly used to check the accuracy of questionnaire translation in cross-cultural studies [44]. Ideally this would briefly imply for the present study that: *i)* a trilingual translator (a competent veterinarian with expertise in the field) translates the questionnaire from the original, source language (here: English) into the two target languages (here: Dutch and Thai); *ii)* another trilingual translator (again a competent veterinarian with expertise in the field) translates the two versions back into English; *iii)* the three English versions of the questionnaire (original and two back-translated versions) are compared for equivalence; *iv)* items with apparent discrepancies between the three English versions are then modified; *v)* the translation/back-translation process is repeated by individual trilingual translators until the original and back-translated versions agreed.

In our study this wasn’t done because at that time trilingual translators, who are veterinarians with appropriate knowledge of the topic, could not be found due to time constrains (if they exist at all). The translation of the English questionnaire to the Thai and Dutch language was done by native speakers in Thai and Dutch who were fluent in English, but not by people who were strictly bilingual as advised by Chen and Boore [33]. They recommend that “the translator is fluent in both the source language and target language and is knowledgeable about both cultures”. Although the translators knew about the human-animal bond and/or had knowledge about the foreign language, the use of non-bilingual translators could have influenced the validity of the questionnaire [34]. However the questionnaires were satisfactorily pre-tested in the target population, which tends to support their validity [34]. But at this stage it is advisable to use the translated questionnaires cautiously.

A possibility would have been to split the results into a Thai part and a Dutch part and describe and discuss the results separately. By taken the Thai and Dutch samples together there is a more powerful experimental design (two-factor design) than with a one-factor design per culture. For this reason and because culture comparisons are interesting, it was decided to do not split the results.

For future cross-cultural studies (with original English questionnaires) involving two non-English speaking countries (e.g. the Netherlands and an Asiatic country) an alternative translation technique might be advisable: the decentering approach [45]. This is a process in which (bilingual) translators move back and forth amongst the languages, checking for cultural and linguistic accuracy. In this way we may prevent that people from the East and the West interpret the questions in different ways because of semantics and cultural differences, when answering questions. It is possible that dog owners in Thailand gave more social desirable answers than owners in the Netherlands. However the translation into Thai and Dutch was done by people living in these cultures.

### Likert scales versus visual analogue scales

The Likert scales used in the original questionnaires of Schneider et al. [12] (5-point scale) and Rohlf et al. [6] (7-point scale) provide ordinal data and so non-parametric tests should be used, which have less power than parametric tests. Furthermore, ordinal scales limit the choice of subsequent (non-parametric) statistical analyses. For this reason, together with the ability to calculate z-scores and perform standard linear principal component analyses, we preferred to use continuous scales (visual analogue scales; see Additional file 1: Appendix, parts 3 and 4). Few studies have compared itemized rating scales with continuous rating scales [46–49], but on the basis of these studies we feel that using a continuous rating scale or a Likert type scale does not affect validity. For example, Lange & Söderlund [46] reported that there were no systematic differences between the two scales. However, changing the scale of a questionnaire from categorical to continuous is not without consequences*.* While McKelvie [49] found that respondents preferred continuous rating scales, these scales were more tiring to use, which could influence the results. McKelvie commented that with smaller numbers of categories there is a loss of discriminative power and values for *r* in correlations, but that with larger numbers of categories, as in our study, a continuous measure does not have psychometric disadvantages [49]. For the moment, there is not enough evidence to conclude that one scale type is psychometric better than the other [50]. Thus we feel, based on Mckelvie [49], that changing the original rating scale of Schneider et al. [12] and Rolhf et al. [6] to a continuous rating scale did not have psychometric consequences. Despite changing the score scale, after a principal axis factoring extraction with varimax rotation (like Schneider et al. [12]), we found a similar magnitude of (absolute) factor loadings for QoL as in Study 2 (ill dogs) of Schneider et al. [12] (Additional file 1: Table S7), suggesting that part 3 of the questionnaire (Additional file 1: Appendix) can also be used by the owners of overweight or obese dogs and is not dependent on the cultural background of the respondent. This probably reflects the fact that obesity is a prevalent illness [22], and that animals with overweight are more likely to become obese. The factor loadings in Study 1 (healthy dogs) were higher than those in Study 2 (ill dogs) in the publication of Schneider et al. [12], and higher than those in our study (paired Student’s *t* test, *P* < 0.005683*). However, the inferential statistical results from Additional file 1: Table S7 should be interpreted with caution. In Schneider et al. [12], items were rated using a 5-point Likert-type scale whereas we used a continuous scale. The most common extraction methods used in factor analysis are principal component analysis and principal axis analysis, which usually result in similar solutions [51]. However, we found that the magnitude of the factor loadings for QoL after principal component analysis were higher than those after principal axis analysis (Additional file 1: Table S7; paired Student’s *t* test, *P* < 0.005683*).

#### Human-dog bond

We did not take the human–animal bond into account because of time restraints – most owners completed the questionnaire while waiting for the veterinarian or waiting for their dogs at a grooming parlour. Although aspects of the human–animal bond influence how owners rate the health of their dogs [12], incorporating these aspects would have made the questionnaire too long, which might have increased the (item) non-response rate, thereby decreasing reliability. The original questionnaire of Schneider et al. [12] was designed for a normal, healthy dog population and an unhealthy, ill dog population in which 7.3% of the dogs were obese.

### QoL

On the basis of owner report, dogs with a BCS of 5 were more immobile than dogs with a BCS of 3, and this effect was more significant in the Dutch cohort than in the Thai cohort (Additional file 1: Table S5). There was no clear association between the z-score for immobility (Additional file 1: Table S5) and the reported total activity (Table 2): for individual respondents (*n =*351) the *R*_*S*_ = -0.045 and the associated *P* = 0.397146 for these two measures. Although we thought that Thai dogs would have less exercise outdoors than the Dutch dogs, this was not the case. It is possible that, in the Netherlands, dogs run free and are not on the leash [52], which might make it difficult for owners to estimate how much exercise their dogs have outdoors. It is also possible that the owners of dogs with a BCS of 4 or 5 gave socially desirable answers, saying that their dogs had a lot of exercise [53].

The observation that owners scored the general sickness component of QoL significantly higher (i.e., worse) when dogs had a higher BCS is surprising, because the dogs that participated in the study were overweight but otherwise healthy. There was also a weak but significant correlation between age of the dog and general sickness (‘dog age’ *vs.* ‘z-score general sickness’: *R*_*S*_ = 0.169, *P* = 0.001442, *n =*354; ‘transformed dog age’ *vs.* ‘transformed z-score general sickness’: *r* = 0.200, *P* = 0.000155, *n =*354). Pearson’s linear correlation coefficient showed that only 4% of the variation in general sickness could be explained by the age of the dog. German et al. [2] reported that QoL improved as dogs lost weight. Psychological, social, and environmental components were not influenced by BCS (Fig. 2), but it is possible that owners found it difficult to assess these QoL aspects.

The questionnaire item (see Additional file 1: Appendix) ‘Anxiety when owner leaves’ maybe synonymous with ‘separation anxiety’. In fact, the term ‘separation-related problems’ is preferable to ‘separation anxiety’ when trying to describe the problematic behaviour seen when a dog is left alone, because the latter implies that a diagnosis has been made [54]. For this reason, we should have used the term ‘separation-related problems’. Even so, the Thai dogs showed more anxiety/separation-related problems than did Dutch dogs when left alone at home. This is difficult to explain but it could be that Thai dogs are less trained or less used to be left alone, possibly because it is usual for Thai households to have a housekeeper or maid, so that there is someone in the house most of the time [55]. Parthasarathy & Crowell-Davis [56] stated that dogs with separation anxiety/separation-related problems may have a different type of attachment relationship with their owners, which makes it more difficult for the dogs to adapt to stressful situations. Mariti et al. [57] showed that not all dog owners are able to correctly address the stress signals of their dogs, which could lead to behaviour problems. Separation anxiety/separation-related behaviour can have different causes [58]. For instance, it is possible that Thai dogs are allowed to roam free to a greater extent than Dutch dogs [18] and hence show greater anxiety/separation-related problems when they are confined to the home alone. But these behaviour problems are not necessarily related to anxiety, and can also reflect relative inactivity [59]. Other QoL scales (social, environmental) were also influenced by culture. Overall, the BCS influenced the physical part of the QoL, and culture had a significant influence on the psychological, social and environmental scales of the QoL.

### Control over feeding and exercise

The questionnaire of Rohlf et al. [6] is based on TPB. It was found that intentions to feed and exercise were best predicted by specific behavioural beliefs or control beliefs, but not by normative beliefs. However, in the original study, Rohlf et al. found only a small proportion of the BCS variance to be explained by feeding behaviours, intentions, and beliefs; none of the exercise variables explained the BCS [6]. We found that the attitude to exercise was an issue for Dutch dog owners and varied significantly between the different BCS groups. This was not the case for the Thai owners (Additional file 1: Table S6).

As the BCS increased, the owners appeared to have less control over feeding and exercise. This is consistent with the findings of Kienzle et al. [10], who reported that the owners of obese dogs believed exercise to be relatively unimportant and that they lacked knowledge about feeding and exercise. In our study, the owners who indicated that they did not exercise their dog enough because they did not like exercising it, had dogs with a higher BCS. The owners of overweight/obese dogs weren’t psychometrically tested of course, but it seems they are more self-centred and put greater emphasis on their own needs than did the owners of normal-weight dogs. Rohlf et al. [6] also found significant correlations between owners’ behavioural beliefs and barriers, normative beliefs and perceptions of control and owners’ behaviour regarding the feeding and exercise of their dogs. Our findings indicate that owner attitudes and beliefs essentially cause obesity because of lack of knowledge and perceived control. This supports the contention of Kienzle et al. [10] that the owners of obese dogs find exercise less important than do the owners of normal weight dogs. Roudebush et al. [60] found that increased physical activity and environmental enrichment are beneficial to overweight dogs and their owners.

In Thailand, dog-centred owners may be influenced by Buddhist beliefs, by which giving food to a dog is good karma. There was a strong cultural influence on the subscale ‘Feed to please’ (Additional file 1: Table S6). Thai owners did this, as was expected, significantly more than did Dutch owners, yet this occurred more often in dogs with low BCS than in dogs with a high BCS, which was unexpected. Dutch dog owners also feed to please, as an expression of their love for their pet [61]. According to Kienzle et al. [10] "owners argue that they love their pets so much that they just can’t deny it treats in the form of food". This belief contributes to overweight and obesity. Further, with a modern life-style, more self-centred owners may have, or take, less time to exercise their dogs [11].

The attitude in feeding of the Dutch dog owners significantly influenced their dog’s BCS whereas this was not the case for the Thai owners (Fig. 3, panel A). This might be because Thai and Dutch owners have a different, culturally determined, bond with their dogs.

## Conclusions

In this study, the Likert-scale questionnaires designed by Schneider et al. [12] and Rohlf et al. [6] were transformed into continuous scales (visual analogue scales), which enabled more powerful statistical techniques to be used and the calculation of integrated z-scores. The magnitude of the factor loadings was similar to that of Study 2 (ill dogs) of Schneider et al. [12]. On the basis of owner-reported information, the QoL of overweight and obese dogs was mainly influenced by the physical status of the dog, whereas psychological aspects of QoL were influenced more by culture (Thai or Dutch) than by BCS. In the Netherlands, owner attitudes to feeding and exercise significantly influenced the BCS. As there were differences on these subscales between the two countries, dog owners in these two countries would appear to have significantly different attitudes towards feeding and exercise. The only exception was when owners seemed to be more self-centred and had less control over feeding, in which case the BCS was increased in dogs in both countries. Obesity in pets is influenced by owner attitudes regarding feeding and exercise, and it is very well possible that the same attitudes influence the health status of the owners. This could explain why there is a correlation between obesity in owners and their dogs [21]. Obesity in both humans and dogs involves a complex multiplicity of genetic and environmental factors [62]. The One Health approach can help to unravel these factors [22].

## Additional file

Additional file 1: **Table S1.** Owner demographics and dog food information: categorical data, **Table** S2 Dog activities: continuous data, **Table S3.** Orthogonal factor loadings per scale for owner-reported quality of life. **Table S4.** Orthogonal factor loadings per scale for owner attitude, **Table S5.** Owner-reported quality of life: subscale z-scores, **Table S6.** Owner attitude: subscale z-scores. **Table S7.** Meaningful comparisons of orthogonal factor loadings for quality of life from this study and the two studies of Schneider et al. [12]. **Figure S1.** Association between years of dog ownership and dog age. Panel A, Thailand; panel B, the Netherlands. **Figure S2.** Association between body condition score and dog age. **Appendix.** the questionnaire. (DOCX 283 kb)

## Abbreviations

ANCOVA: Analysis of covariance
ANOVA: Analysis of variance
BCS: Body condition score
KU-VTH: Kasetsart University Veterinary Teaching Hospital
PAA: Principal axis analysis
PCA: Principal component analysis
QoL: Quality of Life
SD: Standard deviation
SEM: Standard error of the mean
TPB: Theory of planned behaviour
