# Supplementary material for: Quality of life and owner attitude to dog overweight and obesity in Thailand and the Netherlands
Source: BMC Vet Res. 2018 Jul 9;14:221. doi: 10.1186/s12917-018-1531-z (PMC6038310; doi:10.1186/s12917-018-1531-z)
Supplement: Supplementary file 1 — Table S1. Owner demographics and dog food information: categorical data, Table S2 Dog activities: continuous data, Table S3. Orthogonal factor loadings per scale for owner-reported quality of life. Table S4. Orthogonal factor loadings per scale for owner attitude, Table S5. Owner-reported quality of life: subscale z-scores, Table S6. Owner attitude: subscale z-scores. Table S7. Meaningful comparisons of orthogonal factor loadings for quality of life from this study and the two studies of Schneider et al. [12]. Figure S1. Association between years of dog ownership and dog age. Panel A, Thailand; panel B, the Netherlands. Figure S2. Association between body condition score and dog age. Appendix. the questionnaire. (DOCX 283 kb) [file 12917_2018_1531_MOESM1_ESM.docx]

**Supplemental data**

**Table S1** **Owner demographics and dog food information: categorical data^1^**

Body condition score (BCS)

Country: The Netherlands Thailand

Measure *(number of answers)* Log linear analysis

/ *Category* BCS 3 BCS 4 BCS 5 BCS 3 BCS 4 BCS 5 significance**^3^**

Owner? (*n* = 355)^5^

*Owner* 65(97.0%)**^2^** 57(91.9%) 24(92.3%) 64(97.0%) 65(97.0%) 64(95.5%)

*Child of owner* 2(3.0%) 4(6.5%) 1(3.8%) 1(1.5%) 2(3.0%) 3(4.5%)

*Caretaker* 0(0.0%) 1(1.6%) 1(3.8%) 1(1.5%) 0(0.0%) 0(0.0%) C,B,M,CxB

Owner gender (*n* = 355)

*Male* 16(23.9%) 18(29.0%) 8(30.8%) 22(33.3%) 22(32.8%) 20(29.9%)

*Female*  51(76.1%) 44(71.0%) 18(69.2%) 44(66.7%) 45(67.2%) 47(70.1%) C,B,M.CxB

Education level (*n* = 353*) ^A4^ ^B^ ^C^ ^A^ ^B^ ^C^

*Undergraduate secondary school* 0(0.0%) 1(1.6%) 1(3.8%) 5(7.7%) 3(4.5%) 3(4.5%)

*Graduate secondary school –*

*Undergraduate university* 25(37.3%) 34(55.7%) 15(57.7%) 12(18.5%) 11(16.4%) 11(16.4%)

*Graduate university or above* 42(62.7%) 26(42.6%) 10(38.5%) 48(73.8%) 53(79.1%) 53(79.1%) C,B,M,CxB,MxC

**[Table S1, continued]**

Marital status (*n* = 354*) ^A^ ^B^ ^A^ ^B^

*Single* 17(25.3%) 19(30.6%) 12(46.2%) 43(66.2%) 42(62.7%) 47(70.1%)

*Married* 48(71.6%) 41(66.1%) 12(46.2%) 20(30.8%) 25(37.3%) 20(29.9%)

*Divorced* 2(3.0%) 2(3.2%) 2(7.7%) 2(3.1%) 0(0.0%) 0(0.0%) C,B,M,CxB,MxC

Having children (*n* = 355) ^A^ ^B^ ^A^ ^B^

*No* 35(52.2%) 29(46.8%) 13(50.0%) 47(71.2%) 52(77.6%) 56(83.8%)

*Yes* 32(47.8%) 33(53.2%) 13(50.0%) 19(28.8%) 15(22.4%) 11(16.4%) C,B,M,CxB,MxC

Dog food (*n* = 349*) ^A^ ^B^ ^A^ ^B^

*Commercial diet* 65(97.0%) 55(96.5%) 22(88.0%) 53(80.3%) 44(65.7%) 52(77.6%)

*Home-cooked diet* 2(3.0%) 2(3.5%) 3(12.0%) 13(19.7%) 23(34.3%) 15(22.4%) C,B,M,CxB,MxC

**^1^** This table is based on *part 1* and *part 2* of the questionnaire (see Supplementary Appendix).

**^2^** Results are presented as scores (number of cases) with in parentheses the relative frequency (%).

**^3^** Significance (*P* < 0.05) based on log linear analysis with categorical variables/factors *measure* (first column in this table), *country* and *body condition score*. C indicates significant contribution of the factor *country* to the log linear model; B, significant contribution of the factor *body condition score*; CxB, significant contribution of the interaction between the factors *country* and *body condition score*; MxB, significant contribution of the interaction between the variable *measure* and factor *body condition score*; MxC, significant contribution of the interaction between the variable *measure* and factor *country*.

**^4^** Contrast significance (*post hoc* comparisons, *P* < 0.005683). *Post hoc* testing was done by Fischer’s Exact test. Within the same country values with the same superscript lowercase letter were significantly different. Within the same category of body condition score values with the same superscript uppercase letter were significantly different.

**^5^** The number of answers is given in parentheses.

**^*^** Indicates missing answers.

**Table S2** **Dog activities: continuous data^1^**

Measure Body condition score (BCS)

(number of *Transformation* /

answers)**^5^** Country BCS 3 BCS 4 BCS 5 ANOVA significance**^3^**

Indoor activity (h/week)

*Walk* (*n* = 354*)

Netherlands 2.9±0.7^2A^ 1.6±0.5^B^ 0.6±0.4^C4^ *Ranking*

Thailand 25.5±2.9^A^ 24.0±3.2^B^ 22.9±3.6^C^ C

*Run* (*n* = 354*)

Netherlands 0.6±0.2^A^ 0.5±0.2^B^ 0.5±0.3^C^ *Ranking*

Thailand 11.1±1.9^aA^ 6.9±1.0^B^ 5.3±1.0^aC^ C

*Play* (*n* = 353*)

Netherlands 5.4±0.9^aA^ 3.3±0.6^B^ 2.6±1.2^aC^ *Ranking*

Thailand 11.4±1.5^A^ 10.4±1.5^B^ 11.5±2.5^C^ C,B

Outdoor activity (h/week)

*Walk* (*n* = 353*)

Netherlands 10.8±0.8^A^ 10.9±1.0^B^ 16.1±3.6^C^ *Ranking*

Thailand 9.8±1.7^A^ 6.3±1.3^B^ 9.1±2.5^C^ C,B,CxB

*Run* (*n* = 354*)

Netherlands 3.9±0.5 3.7±0.6 2.7±0.8 *Ranking*

Thailand 6.5±1.1 4.1±0.8 5.2±1.3 C,B,CxB

*Play* (*n* = 354*)

Netherlands 5.0±1.0^ab^ 3.7±1.1^a^ 1.9±0.7^b^ *Ranking*

Thailand 4.7±1.1 2.7±0.6 6.1±2.0 C,B,CxB

Indoor plus outdoor activity (h/week)

*Walk* (*n* = 353*)

Netherlands 13.6±1.2^A^ 12.5±1.0^B^ 16.8±3.6 *Ranking*

Thailand 35.3±3.0^A^ 30.3±3.2^B^ 32.3±4.4 C

*Run* (*n* = 354*)

Netherlands 4.6±0.6^A^ 4.2±0.7^B^ 3.2±1.0^C^ *Ranking*

Thailand 17.6±2.4^aA^ 11.0±1.4^B^ 10.5±1.8^aC^ C,B,CxB

*Play* (*n* = 353*)

Netherlands 10.5±1.6^a^ 7.0±1.5^A^ 4.5±1.8^aB^ *y = ^10^Log[x + 2.0]*

Thailand 16.1±2.2 13.1±1.8^A^ 17.7±3.9^B^ C,B

**^1^** This table is based on *part 1* and *part 2* of the questionnaire (see Supplementary Appendix).

**[Table S2, continued]**

**^2^** Results are presented as means ± SEM.

**^3^** Significance (*P* < 0.05) based on two-way ANOVA with main factors *country* and *body condition score*. C indicates effect of *country*; B, effect of *body condition score*; CxB, interaction. Measures that are not normally distributed and/or where the variances were unequal, were first transformed. In this column the type of transformation is also given.

**^4^** Contrast significance (*post hoc* comparisons, *P* < 0.005683). *Post hoc* testing was done by unpaired Student’s *t* test (Gaussian distributed data + homoscedasticity), unpaired Student’s *t* test with Welch-Satterthwaite correction (Gaussian distributed data + heteroscedasticity) or Wilcoxon-Mann-Whitney test (non-Gaussian distributed data). Within the same row (country) values with the same superscript lowercase letter were significantly different. Within the same column (body condition score) values with the same superscript uppercase letter were significantly different.

**^5^** The number of answers is given in parentheses.

**^*^** Indicates missing answers.

**Table S3** **Orthogonal factor loadings per scale for owner-reported quality of life^1^**

Scale / Orthogonal factors

*Subscale* / GS IM IM/EI GA/AL AL DF SO SA/BN BN

Question number Item Factor: Phy1 Phy2 Phy3 Psy1 Psy2 Soc1 Soc2 Env1 Env2

Eigenvalue 4.22 1.94 1.56 2.45 1.15 2.12 1.23 1.47 1.02

% of the total variance 23.4 10.8 8.7 40.7 19.1 35.4 20.5 36.7 25.6

Physical (Phy)

(Kaiser-Meyer-Olkin measure: 0.772)

*General sickness (GS)*

Q1: *My dog acts sick.* **0.574** 0.182 0.183 – – – – – –

Q2: *My dog does not like being touched.* **0.532** 0.135 -0.178 – – – – – –

Q3: *My dog has difficulty eliminating or eliminates more frequently than*

*usual*. **0.608** 0.191 -0.031 – – – – – –

Q4: *My dog has difficulty sleeping.* **0.624** 0.020 0.006 – – – – – –

Q5: *My dog vomits more than he/she used to.* **0.560** -0.080 0.123 – – – – – –

Q6: *My dog has difficulty breathing.* **0.411** 0.383 0.271 – – – – – –

Q7: *My dog’s temperament has changed.* **0.671** 0.215 0.051 – – – – – –

Q8: *My dog has become aggressive to dogs or people who were accepted*

*before.* **0.472** -0.039 0.312 – – – – – –

Q9: *My dog gets lost in places that should be familiar.* 0.393 0.172 0.210 – – – – – –

Q10: *My dog shows less desire to interact with other dogs or people than*

*usual.* **0.406** 0.249 0.297 – – – – – –

*Immobility (IM)*

**[Table S3, continued]**

Q11: *My dog has a lot of energy.* 0.016 -0.089 **-0.645** – – – – – –

Q12: *My dog rarely gets excited anymore.* 0.085 **0.590** 0.017 – – – – – –

Q13: *My dog has difficulty getting up after lying down.* 0.075 **0.850** 0.063 – – – – – –

Q14: *My dog has difficulty walking.* 0.128 **0.842** 0.042 – – – – – –

Q15: *My dog plays less often.* 0.158 **0.720** -0.006 – – – – – –

Q16: *My dog’s overall mobility is good (reverse score).* 0.071 0.016 **-0.764** – – – – – –

*External irritation (EI)*

Q17: *My dog chews or scratches certain areas of skin until they become red*

*and irritated.* 0.143 0.019 **0.610** – – – – – –

Q18: *My dog has patches of fur missing.* 0.293 0.029 **0.497** – – – – – –

Psychological (Psy)

(Kaiser-Meyer-Olkin measure: 0.683)

*Anxiety when owner leaves (AL)*

Q19: *My dog chews on things that he/she has been taught not to do.* – – – 0.019 **0.858** – – – –

Q20: *My dog whines when I leave.* – – – **0.516** 0.277 – – – –

Q21: *My dog makes a mess when I’m away from home.* – – – 0.272 **0.811** – – – –

*General anxiety (GA)*

Q22: *My dog is startled easily.* – – – **0.746** 0.101 – – – –

Q23: *My dog cowers when it meets a new person or dog.* – – – **0.797** 0.011 – – – –

Q24: *When my dog is in a new place, it puts its tail between its legs.* – – – **0.742** 0.160 – – – –

Social (Soc)

(Kaiser-Meyer-Olkin measure: 0.652)

*Dog focused (DF)*

**[Table S3, continued]**

Q25: *I play with my dog when it is ready to play.* – – – – – **0.437** 0.104 – –

Q26: *I pet my dog often.* – – – – – **0.800** 0.006 – –

Q27: *I groom my dog often.*  – – – – – **0.713** 0.099 – –

Q28: *I often spend my free time with my dog.* – – – – – **0.736** 0.113 – –

*Sociability (SO)*

Q29: *My dog has the opportunity to play with other dogs.* – – – – – 0.091 **0.850** – –

Q30: *My dog shares his/her toys with other animals.* – – – – – 0.124 **0.834** – –

Environmental (Env)

(Kaiser-Meyer-Olkin measure: 0.569)

*Basic needs (BN)*

Q31: *My dog has fresh water available throughout the day.* – – – – – – – **0.628** 0.288

Q32: *My dog goes outside when he/she needs to.*  – – – – – – – -0.024 **0.946**

*Sleeping area (SA)*

Q33: *My dog’s sleeping area is her/her own.* – – – – – – – **0.775** -0.017

Q34: *My dog has his/her own place to sleep (e.g. bed of the owner).* – – – – – – – **0.687** -0.211

**^1^** The data from the owner reports of quality of life (*n* = 355) were subject to several factor analyses. The Kaiser-Meyer-Olkin measures were high (should be > ~0.5), indicating a high sampling adequacy for the factor analyses. Bartlett’s test of sphericity indicates that the factor model is appropriate (*P* < 0.0005). Factor loadings > 0.4 are considered to be high and are indicated in bold. GS = general sickness, IM = immobility, EI = external irritation, AL = anxiety when owner leaves, GA = general anxiety, DF = dog focused, SO = sociability, BN = basic needs, SA = sleep area.

**Table S4** **Orthogonal factor loadings per scale for owner attitude^1^**

Orthogonal factors

Scale / OE FP OC VE

*Subscale* / DB AK DB FCB AK OC ECB LK EB VE DC

Question number Item Factor: Afe1 Afe2 Afe3 Afe4 Afe5 Aex1 Aex2 Aex3 Aex4 Aex5 Aex6

Eigenvalue 6.23 1.69 1.54 1.42 1.18 6.67 3.24 2.29 1.67 1.61 1.50

% of the total variance 34.6 9.4 8.6 7.9 6.5 27.8 13.5 9.5 7.0 6.7 6.3

Attitude in feeding (Afe)

(Kaiser-Meyer-Olkin measure: 0.832)

*Ambivalence about knowledge (AK)*

Q35: *I don’t know how much food to feed my dog.* 0.219 **0.757** 0.043 -0.181 -0.215 – – – – – –

Q36: *I don’t know what type of food to feed my dog.* 0.097 **0.823** 0.109 -0.174 -0.144 – – – – – –

Q37: *I don’t know how many times a day I should feed my dog.* 0.112 **0.807** 0.093 -0.171 -0.027 – – – – – –

Q38: *It’s important that I feed my dog the appropriate type of food.*-0.079 -0.163 -0.164 0.162 **0.882** – – – – – –

Q39: *It’s important that I feed my dog the appropriate number of*

*times a day.* -0.144 -0.171 -0.220 0.088 **0.875** – – – – – –

*Feed to please (FP)*

Q40: *It’s important that I feed my dog whenever he/she likes.* 0.099 0.006 **0.727** -0.178 -0.157 – – – – – –

Q41: *It’s important that I feed my dog whatever he/she likes.* 0.189 0.150 **0.787** -0.095 -0.016 – – – – – –

Q42: *It’s important that I feed my dog as much as he/she wants.* 0.089 0.070 **0.782** -0.122 -0.298 – – – – – –

*Owner-centred / External barrier (OE)*

Q43: *My dog is overfed because he/she always wants food.* **0.562** 0.182 0.317 -0.204 0.041 – – – – – –

**[Table S4, continued]**

Q44: *My dog isn’t given the appropriate type of food because*

*others feed the dog.* **0.620** -0.160 -0.097 -0.347 -0.178 – – – – – –

Q45: *I feed my dog inappropriate types of food because he/she*

*likes that kind of food.* **0.686** 0.177 0.114 -0.113 -0.210 – – – – – –

Q46: *My dog isn’t fed the appropriate number of times per day*

*because others feed him/her.* **0.665** -0.066 -0.152 -0.267 -0.180 – – – – – –

*Dog-centred barriers (DB)*

Q47: *I feed my dog inappropriate food because I like to spoil*

*him/her.* **0.685** 0.316 0.293 0.017 -0.074 – – – – – –

Q48: *My dog is overfed because I indulge him/her.* **0.603** 0.319 **0.405** -0.150 0.024 – – – – – –

Q49 : *I feed my dog inappropriate food because appropriate food*

*is too expensive.* **0.513** 0.188 0.253 -0.081 0.150 – – – – – –

*Control belief (FCB)*

Q50: *Overall, how much control do you feel you have over the*

*amount you feed your dog.* -0.188 -0.186 -0.248 **0.791** 0.145 – – – – – –

Q51: *Overall, how much control do you feel you have over the*

*type of food you feed your dog.* -0.192 -0.196 -0.172 **0.811** 0.111 – – – – – –

Q52: *Overall, how much control do you feel you have over the*

*number of times you feed your dog during the day.* -0.246 -0.239 -0.094 **0.751** 0.045 – – – – – –

Attitude in exercise (Aex)

(Kaiser-Meyer-Olkin measure: 0.828)

*Value exercise (VE)*

Q53: *It’s important that I exercise my dog the appropriate number*

*of times a week.* – – – – – -0.139 -0.145 -0.050 -0.001 **0.837** 0.076

**[Table S4, continued]**

Q54: *It’s important that I give my dog the appropriate type of*

*exercise.*  – – – – – -0.111 -0.081 -0.158 -0.066 **0.846** 0.017

Q55: *It’s important to me that my dog is fit.* – – – – – -0.089 0.063 -0.019 0.008 **0.720** 0.122

Q56: *My dog doesn’t need exercise.* – – – – – -0.042 0.299 **0.516** 0.022 0.154 0.203

Q57: *It is important that I exercise my dog for the appropriate*

*length of time.* – – – – – -0.100 0.071 0.248 -0.045 **0.546** 0.158

*Lack of knowledge (LK)*

Q58: *I don’t know how often I should exercise my dog.*  – – – – – 0.182 0.208 **0.841** 0.099 -0.065 0.084

Q59: *I don’t know the appropriate length of time my dog should*

*be exercised.* – – – – – 0.195 0.221 **0.868** 0.101 -0.082 0.063

Q60: *I don’t know what type of exercise to give my dog*. – – – – – 0.210 0.156 **0.873** 0.083 -0.021 0.054

*Dog centred (DC)*

Q61: *It’s important that I exercise my dog as frequently*

*as he/she wants.* – – – – – 0.009 0.153 0.155 0.020 0.147 **0.816**

Q62: *It’s important that I exercise my dog for as long as*

*he/she wants.*  – – – – – 0.033 0.039 0.090 -0.038 0.074 **0.873**

Q63: *It’s important that I give my dog the type of exercise that*

*he/she likes.* – – – – – -0.021 0.054 0.034 0.066 0.122 **0.804**

*Owner centred (OC)*

Q64: *I don’t exercise my dog frequently enough because I don’t*

*like to.* – – – – – **0.855** 0.126 0.152 0.031 -0.113 -0.014

Q65: *I don’t exercise my dog for long enough because I don’t*

*like to.* – – – – – **0.870** 0.127 0.135 0.081 -0.113 0.039

Q66: *I don’t give my dog the appropriate type of exercise*

*because I don’t like to.* – – – – – **0.825** 0.174 0.114 0.069 -0.139 0.010

**[Table S4, continued]**

Q67: *I don’t give my dog the appropriate kind of exercise*

*because he/she doesn’t like that type of exercise.* – – – – – **0.644** 0.084 0.142 0.139 -0.225 0.098

Q68: *I don’t exercise my dog as frequently as I should because*

*he/she is badly behaved.* – – – – – **0.440** 0.141 -0.075 0.290 0.012 -0.110

Q69: *I don’t exercise my dog as frequently as I should because*

*I don’t have time.* – – – – – **0.486** **0.499** 0.189 0.135 -0.003 -0.037

Q70: *I don’t give my dog the appropriate type of exercise*

*because I don’t have access to appropriate areas.* – – – – – 0.319 **0.489** 0.239 0.051 0.112 -0.081

*External barrier (EB)*

Q71: *My dog isn’t exercised frequently enough because other*

*people exercise it.* – – – – – 0.135 0.113 0.075 **0.921** -0.006 -0.004

Q72: *My dog isn’t given the appropriate type of exercise*

*because other people exercise it.* – – – – – 0.119 0.075 0.109 **0.927** -0.057 0.054

Q73: *My dog isn’t exercised long enough because other people*

*exercise it.* – – – – – 0.151 0.084 0.093 **0.914** -0.041 0.028

*Control belief (ECB)*

Q74: *Overall, how much control do you feel you have over the*

*type of exercise you give your dog.* – – – – – -0.146 **-0.848** -0.196 -0.074 0.077 -0.109

Q75: *Overall, how much control do you feel you have over how*

*frequently you exercise your dog.* – – – – – -0.182 **-0.894** -0.184 -0.098 0.041 -0.145

Q76: *Overall, how much control do you feel you have over the*

*length of time you exercise your dog?* – – – – – -0.123 **-0.899** -0.202 -0.101 0.067 -0.130

**[Table S4, continued]**

^1^ The data from the owner reports of quality of life (*n* = 355) were subject to several factor analyses. The Kaiser-Meyer-Olkin measures were high (should be > ~0.5), indicating a high sampling adequacy for the factor analysis. Bartlett’s test of sphericity indicates that the factor model is appropriate (*P* < 0.0005). Factor Loadings > 0.4 are considered to be high and are indicated in bold. The eleven factors account for 72.0% of the total variance. AK = ambivalence about knowledge, FP = feed to please, OE = owner-centred / external barrier, DB = dog-centred barriers, FCB = control belief (part of: attitude of feeding), VE = value exercise, LK = lack of knowledge, DC = dog centred, OC = owner centred, EB = external barrier, ECB = control belief (part of: attitude in exercise).

**Table S5** **Owner-reported quality of life: subscale z-scores^1^**

Scale / *Subscale* Body condition score (BCS)

(number of *Transformation* /

answers)**^6^** Country BCS 3 BCS 4 BCS 5 ANOVA significance**^4^**

*(Subscale z-scores)***^2^**

Physical

*General sickness:***^3^** [ZQ1 + ZQ2 + ZQ3 + ZQ4 + ZQ5 + ZQ6 + ZQ7 + ZQ8 + ZQ9 + ZQ10]/10

(*n* = 354*****) Netherlands -0.32±0.04^abA^**^5^** -0.00±0.08^a^ 0.20±0.14^b^ *y = ^10^Log[x + 0.7]*

Thailand -0.01±0.07^A^ 0.13±0.07 0.11±0.06 C,B,CxB

*Immobility:* [ZQ12 + ZQ13 + ZQ14 + ZQ15 – ZQ11 – ZQ16]/6

(*n* = 354*****) Netherlands -0.33±0.08^aA^ -0.17±0.08^b^ 0.68±0.17^abB^ *Ranking*

Thailand 0.01±0.05^A^ 0.08±0.05 0.13±0.05^B^ C,B,CxB

*External irritation:* [ZQ17 + ZQ18]/2

(*n* = 355) Netherlands -0.50±0.05^abA^ -0.12±0.11^a^ -0.05±0.16^b^ *Ranking*

Thailand 0.12±0.10^A^ 0.26±0.11 0.25±0.11 C,B,CxB

Psychological

*Anxiety when owner leaves:* [ZQ19 + ZQ20 + ZQ21]/3

(*n* = 355) Netherlands -0.37±0.06^A^ -0.27±0.07^B^ -0.12±0.14 *Ranking*

Thailand 0.44±0.11^A^ 0.18±0.08^B^ 0.05±0.08 C,B,CxB

*General anxiety:* [ZQ22 + ZQ23 + ZQ24]/3

(*n* = 355) Netherlands -0.38±0.07^A^ -0.22±0.08^B^ -0.28±0.14 *Ranking*

Thailand 0.31±0.11^A^ 0.30±0.10^B^ 0.09±0.09 C *(C,CxB)*

Social

*Dog focused:* [ZQ25 + ZQ26 + ZQ27 + ZQ28]/4

(*n* = 354*****) Netherlands -0.21±0.09^A^ -0.06±0.08 -0.17±0.17 *No transformation*

Thailand 0.18±0.08^A^ -0.03±0.08 0.18±0.08 C

*Sociability:* [ZQ29 + ZQ30]/2

(*n* = 353*****) Netherlands 0.08±0.11 0.10±0.11 -0.42±0.16 *No transformation*

Thailand -0.03±0.10 -0.00±0.10 0.03±0.10 C *(*–*)*

Environmental

*Basic needs:* [ZQ31 + ZQ32]/2

(*n* = 355) Netherlands 0.08±0.08 0.10±0.10 0.12±0.18 *Ranking*

Thailand 0.08±0.07 -0.21±0.11 -0.08±0.08 C

*Sleeping area:* [ZQ33 + ZQ34]/2

(*n* = 355) Netherlands -0.08±0.09 -0.29±0.13 -0.13±0.17 *Ranking*

Thailand 0.13±0.10 0.11±0.09 0.15±0.08 C *(C,B,CxB)*

**[Table S5, continued]**

**^1^** This table is based on *part 3* of the questionnaire (see Appendix).

**^2^** Results are presented as means ± SEM.

**^3^** For each questionnaire item z-scores for individual respondents were calculated. The z-scores for questionnaire items (here indicated as ZQ1, ZQ2, ZQ3, etc.) were averaged within a subscale according to the given formula, resulting in a subscale z-score.

**^4^** Significance (*P* < 0.05) based on two-way ANOVA with main factors *country* and *body condition score*. C indicates effect of *countr*y; B, effect of *body condition score*; CxB, interaction. Subscale z-scores that are not normally distributed and/or where the variances were unequal, were first transformed. In this column the type of transformation is also given. The data were also tested for significant differences by an ANCOVA with main factors *country* and *body condition score*, *dog’s gender* and *sexual status*. Covariates were *age of the dog* and *duration of ownership*. If the effects in the ANCOVA of the main factors *country*, *body condition score* and their *interaction* were different from those obtained with the two-way ANOVA than this is indicated in parentheses and in italics. – = no significant C, B and CxB effect.

**^5^** Contrast significance (*post hoc* comparisons, *P* < 0.005683). *Post hoc* testing was done by unpaired Student’s *t* test (Gaussian distributed data + homoscedasticity), unpaired Student’s *t* test with Welch-Satterthwaite correction (Gaussian distributed data + heteroscedasticity) or Wilcoxon-Mann-Whitney test (non-Gaussian distributed data). Within the same row (country) values with the same superscript lowercase letter were significantly different. Within the same column (body condition score) values with the same superscript uppercase letter were significantly different.

**^6^** The number of answers is given in parentheses.

**^*^** Indicates missing answers.

**Table S6** **Owner attitude: subscale z-scores^1^**

Scale / *Subscale* Body condition score (BCS)

(number of Transformation /

answers)**^6^** Country BCS 3 BCS 4 BCS 5 ANOVA significance**^4^**

*(Subscale z-scores)***^2^**

Attitude in feeding

*Ambivalence about knowledge:***^3^** [ZQ35 + ZQ36 + ZQ37 – ZQ38 – ZQ39]/5

(*n* = 354*****) Netherlands -0.74±0.04^aA^**^5^** -0.53±0.07^B^ -0.26±0.16^aC^ *Ranking*

Thailand 0.34±0.07^A^ 0.53±0.07^B^ 0.44±0.06^C^ C,B,CxB

*Feed to please:* [ZQ40 + ZQ41 + ZQ42]/3

(*n* = 355) Netherlands -0.56±0.08^aA^ -0.29±0.08^aB^ -0.23±0.16 *Ranking*

Thailand 0.45±0.10^A^ 0.21±0.09^B^ 0.26±0.11 C,B,CxB

*Owner-centred / External barrier:* [ZQ43 + ZQ44 + ZQ45 + ZQ46]/4

(*n* = 355) Netherlands -0.48±0.05^abA^ -0.19±0.06^aB^ 0.03±0.14^b^ *Ranking* Thailand 0.07±0.09^A^ 0.31±0.10^B^ 0.27±0.10 C,B,CxB

*Dog-centred barriers:* [ZQ47 + ZQ48 + ZQ49]/3

(*n* = 354*****) Netherlands -0.50±0.06^abA^ -0.16±0.10^aB^ 0.19±0.20^b^ *Ranking* Thailand 0.04±0.09^A^ 0.30±0.10^B^ 0.23±0.10 C,B,CxB

*Control belief:* [ZQ50 + ZQ51 + ZQ52]/3

(*n* = 355) Netherlands 0.69±0.08^abA^ 0.11±0.15^aB^ 0.13±0.15^bC^ *Ranking* Thailand -0.10±0.09^cdA^ -0.47±0.09^cB^ -0.48±0.10^dC^ C,B,CxB

Attitude in exercise

*Value of exercise:* [ZQ53 + ZQ54 + ZQ55 – ZQ56 + ZQ57]/5

(*n* = 354*****) Netherlands 0.20±0.09^aA^ 0.07±0.08 -0.34±0.15^a^ *Ranking* Thailand -0.06±0.07^A^ -0.08±0.06 0.01±0.06 C,B,CxB

*Lack of knowledge:* [ZQ58 + ZQ59 + ZQ60]/3

(*n* = 354*****) Netherlands -0.68±0.08^aA^ -0.55±0.09^B^ -0.12±0.19^a^ *Ranking* Thailand 0.20±0.11^A^ 0.60±0.11^B^ 0.44±0.09 C,B,CxB

*Dog centred:* [ZQ61 + ZQ62 + ZQ63]/3

(*n* = 355) Netherlands -0.33±0.11^A^ -0.36±0.12^B^ -0.32±0.18 *No transformation*

Thailand 0.35±0.09^A^ 0.21±0.09^B^ 0.23±0.08 C

*Owner centred:* [ZQ64 + ZQ65 + ZQ66 + ZQ67 + ZQ68 + ZQ69 + ZQ70]/7

(*n* = 355) Netherlands -0.43±0.05^abA^ -0.17±0.06^aB^ 0.10±0.13^b^ *y = ^10^Log[x + 1.0]*

Thailand 0.02±0.10^A^ 0.24±0.10^B^ 0.29±0.09 C,B

*External barrier:* [ZQ71 + ZQ72 + ZQ73]/3

(*n* = 355) Netherlands -0.34±0.06 -0.08±0.16 -0.08±0.16 *Ranking*

Thailand 0.03±0.14 0.12±0.12 0.30±0.14 C,B,CxB

**[Table S6, continued]**

*Control belief:* [ZQ74 + ZQ75 + ZQ76]/3

(*n* = 353*****) Netherlands 0.75±0.06^A^ 0.42±0.12^B^ 0.31±0.18^C^ *Ranking*

Thailand -0.23±0.11^A^ -0.49±0.11^B^ -0.52±0.09^C^ C,B,CxB

**^1^** This table is based on *part 4* of the questionnaire (see Appendix).

**^2^** Results are presented as means ± SEM.

**^3^** For each questionnaire item z-scores for individual respondents were calculated. The z-scores for questionnaire items (here indicated as ZQ35, ZQ36, ZQ37, etc.) were averaged within a subscale according to the given formula, resulting in a subscale z-score.

**^4^** Significance (*P* < 0.05) based on two-way ANOVA with main factors country and body condition score. C indicates effect of country; B, effect of body condition score; CxB, interaction. Subscale z-scores that are not normally distributed and/or where the variances were unequal, were first transformed. In this column the type of transformation is also given. The data were also tested for significant differences by an ANCOVA with main factors *country* and *body condition score*, *dog’s gender* and *sexual status*. Covariates were *age of the dog* and *duration of ownership*. If the effects in the ANCOVA of the main factors *country*, *body condition score* and their *interaction* were different from those obtained with the two-way ANOVA than this is indicated in parentheses and in italics. – = no significant C, B and CxB effect.

**^5^** Contrast significance (*post hoc* comparisons, *P* < 0.005683). *Post hoc* testing was done by unpaired Student’s *t* test (Gaussian distributed data + homoscedasticity), unpaired Student’s *t* test with Welch-Satterthwaite correction (Gaussian distributed data + heteroscedasticity) or Wilcoxon-Mann-Whitney test (non-Gaussian distributed data). Within the same row (country) values with the same superscript lowercase letter were significantly different. Within the same column (body condition score) values with the same superscript uppercase letter were significantly different.

**^6^** The number of answers is given in parentheses.

**^*^** Indicates missing answers.

**Table S7** **Meaningful comparisons of orthogonal factor loadings for quality of life from this study and the two studies of Schneider et al. [12]^1^**

This study Schneider et al. [12]

Principal components analysis *(PCA)* Principle axis analysis *(PAA)* Principle axis analysis *(PAA)*

Over all scales Per scale Over all scales Per scale Study 1 Study 2

(Table 3) (Table S3) (healthy dogs) (ill dogs)

*Mean ± SEM:***^1^** 0.642±0.022 0.687±0.026 0.547±0.024 0.549±0.032 0.654±0.018 0.581±0.023

This study

*PCA* – *t_29_* = -1.712^2^ *t_29_* = 9.297 – – –

*(Table 3)* *P* = 0.097549 ***P < 0.0000005****

*PCA* – – – *t_29_* = 5.011 – –

*(Table S3)* ***P = 0.000025****

*PAA* – – – *t_29_* = -0.101 *t_29_* = -4.720 *t_29_* = -1.706

*(Over all scales) P* = 0.920591 ***P = 0.000055**** *P* = 0.098769

*PAA* – – – – *t_29_* = -3.431 *t_29_* = -1.252

*(Per scale)* ***P = 0.001829**** *P* = 0.220603

**[Table S7, continued]**

Schneider et al. [12]

*Study 1* *(PAA)* – – – – – *t_29_* = 3.845

***P = 0.000609****

*Study 2 (PAA)* – – – – – –

**^1^** Summarizing values for the factor loadings are based on 30 questions.

**^2^** Results (*t* and *P* value, as well as *df*) from paired Student’s *t* test.

**^*^** Indicates a significant difference (*P* < 0.005683), which is also in ***bolditalics***.

**Figure S1. *Association between years of dog ownership and dog age. Panel A, Thailand; panel B, the Netherlands.***


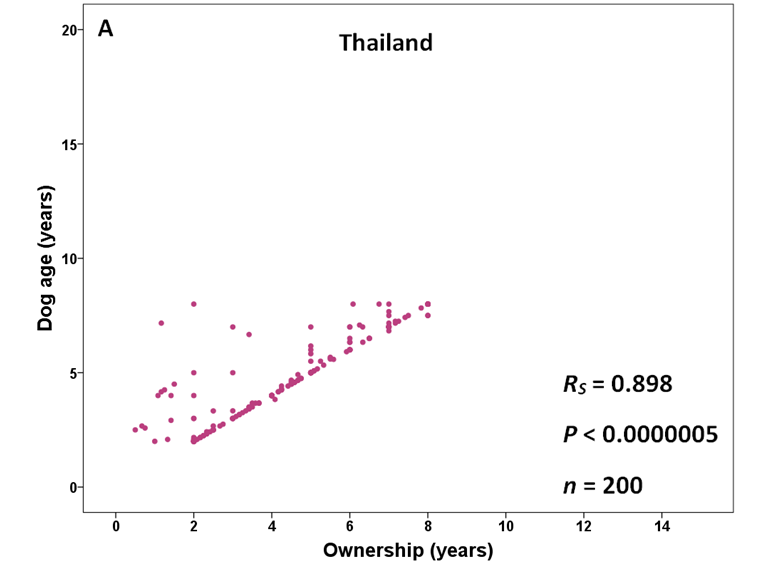


**Figure S1, panel A**


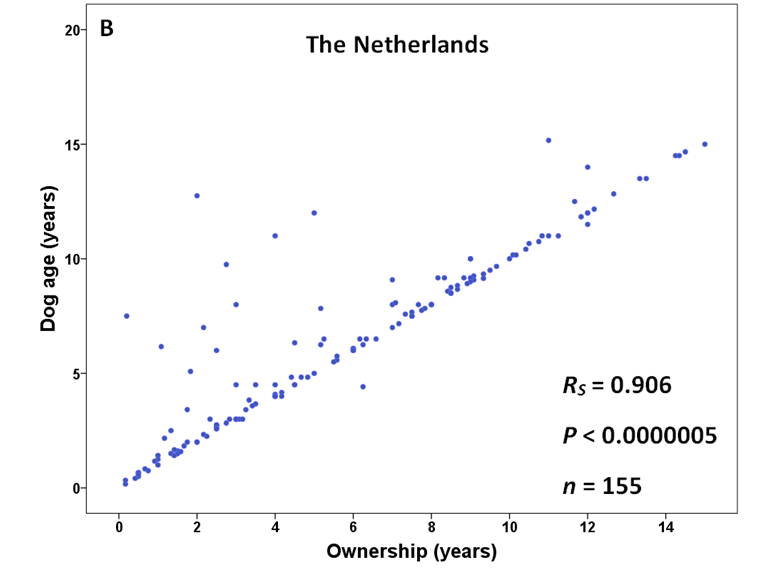


**Figure S1, panel B**

**Figure S2. *Association between body condition score and dog age.***


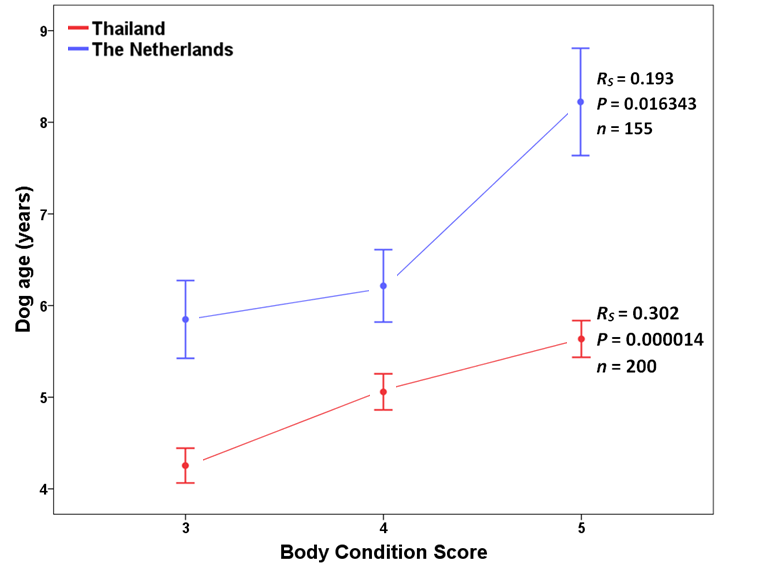


**Figure S2**

**Appendix;** the questionnaire

For staff/personnel: sample number: __ __ __

Body condition score □ 3/5 □4/5 □5/5

**Please** put **X** into the box and fill the information into the blank.

**PART 1: Information of the owner**

Are you the owner of the dog?: □ Yes □ No, I am _________________

(Please indicate relationship)

What is your age? : __________________years

What is your sex?: □ Male □ Female

Level of Education: □ Undergraduate secondary school

□ Graduated secondary school - Undergraduate Bachelor degree

□ Graduated Bachelor - Post Graduated degree

How long have you been the owner or caretaker of this dog?: ________years, ________ months

□ Not applicable

What is your marital status? □single □married □ other: _______________

(Please indicate status)

Do you have any children? □ No

□ Yes, (Please indicate number of children you have and their ages)

- I have __________child/children (Fill in number of children)

- Age of my child (/children):______________________________ Years

(Fill in the age of your child/children)

**Information about the dog**

What is the breed of the dog? : ______________________

What is the dog's gender and sexual status?

□Male neutered □ Male sexually intact

□Female neutered □ Female sexually intact

How old is the dog?: ____________years, __________months

**PART 2:**

What type of food does the dog usually get? (Select type/ please indicate brand or formula)

□ Commercial diet: _________________

□ Homemade diet: _________________

□ Other: ___________________________

How much food is the dog given?

□ As much as it needs (always have the food in the bowl or plate)

□ As indicated on the product bag or according to the guidelines

□ As a crude estimation

□ Do not know

How much exercise does the dog get? (Please fill in the table)

| Indoor activity | Hour(s)/Week | Outdoor activity | Hour(s)/Week |
| --- | --- | --- | --- |
| Walking |  | Walking |  |
| Running |  | Running |  |
| Playing |  | Playing |  |
| Other:_________ |  | Other:_________ |  |
| Other:_________ |  | Other:_________ |  |
| Other:_________ |  | Other:_________ |  |

**Instruction: Please indicate** the extent to what do you agree with the following statement by putting  **∕** somewhere on the line


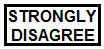


Put  **∕** somewhere on the line to rate your point of view, (only one in each line)

**PART 3:**

| 1. My dog acts sick. | 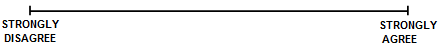 |
| --- | --- |
| 2. My dog does not like being touched. | 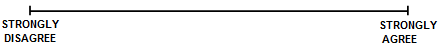 |
| 3. My dog has difficultly eliminating or eliminates more frequently than usual. | 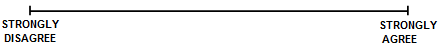 |
| 4. My dog has difficulty sleeping. | 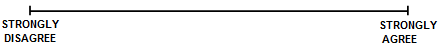 |
| 5. My dog vomits more than he/she used to. | 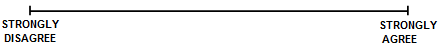 |
| 6. My dog has difficulty breathing. | 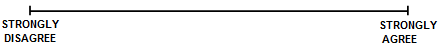 |
| 7. My dog’s temperament has changed. | 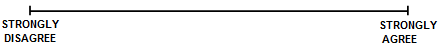 |
| 8. My dog has become aggressive to dogs or people who were accepted before. | 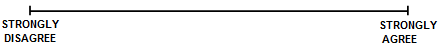 |
| 9. My dog gets lost in places that should be familiar. | 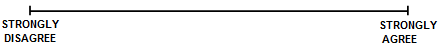 |
| 10. My dog shows less desire to interact with other dogs or people than usual. | 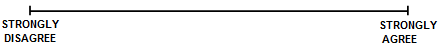 |
| 11. My dog has a lot of energy. | 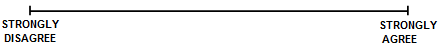 |
| 12. My dog rarely gets excited anymore. | 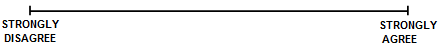 |
| 13. My dog has difficulty getting up after lying down. | 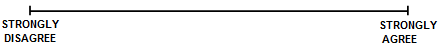 |
| 14. My dog has difficulty walking. | 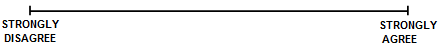 |
|  |  |
| 15. My dog plays less often. | 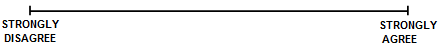 |
|  |  |
| 16. My dog’s overall mobility is good (reverse score). | 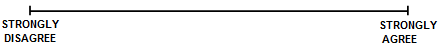 |
| 17. My dog chews or scratches certain areas of skin until they become red and irritated. | 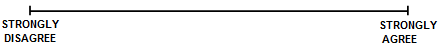 |
| 18. My dog has patches of fur missing. | 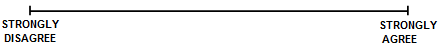 |
| 19. My dog chews on things that he/she has been taught not to do. | 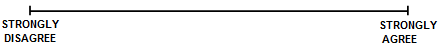 |
| 20. My dog whines when I leave. | 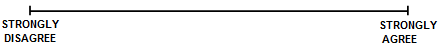 |
| 21. My dog makes a mess when I’m away from home. | 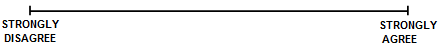 |
| 22. My dog is startled easily. | 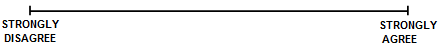 |
| 23. My dog cowers when it meets a new person or dog. | 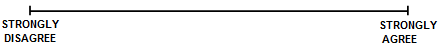 |
| 24. When my dog is in a new place, it puts its tail between its legs. | 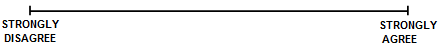 |
| 25. I play with my dog when it is ready to play. | 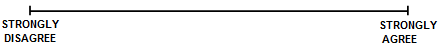 |
| 26. I pet my dog often. | 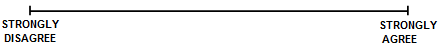 |
| 27. I groom my dog often. | 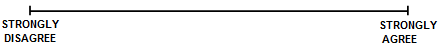 |
| 28. I often spend my free time with my dog. | 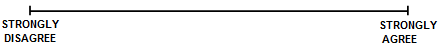 |
| 29. My dog has the opportunity to play with other dogs. | 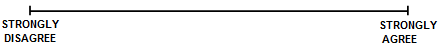 |
| 30 My dog shares his/her toys with other animals. | 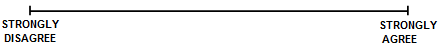 |
| 31. My dog has fresh water available throughout the day. | 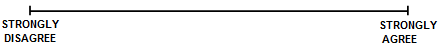 |
| 32. My dog goes outside when he/she needs to. | 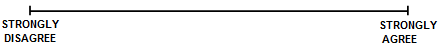 |
| 33. My dog's sleeping area is his/her own. | 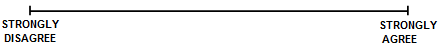 |
| 34. My dog has his/her own place to sleep (e.g. bed of the  owner). | 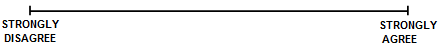 |
|  |  |

**PART 4:**

| 35. I don't know how much food to feed my dog. | 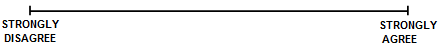 |
| --- | --- |
| 36. I don't know what type of food to feed my dog. | 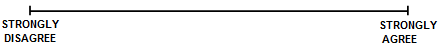 |
| 37. I don't know how many times a day I should feed my dog. | 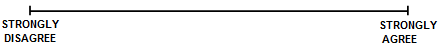 |
| 38. It's important that I feed my dog the appropriate type of food. | 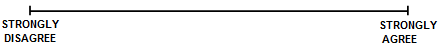 |
| 39. It's important that I feed my dog the appropriate number of times a day. | 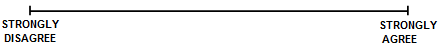 |
| 40. It’s important that I feed my dog whenever he/she likes. | 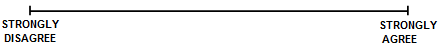 |
| 41. It’s important that I feed my dog whatever he/she likes. | 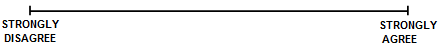 |
| 42. It’s important that I feed my dog as much as he/she wants. | 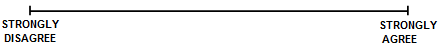 |
| 43. My dog is overfed because he/she always wants food. | 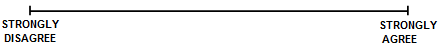 |
| 44. My dog isn’t given the appropriate type of food because others feed the dog. | 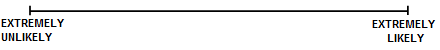 |
| 45. I feed my dog inappropriate types of food because he/she likes that kind of food. | 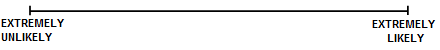 |
| 46. My dog isn’t fed the appropriate number of times per day because others feed him/her. | 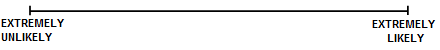 |
| 47. I feed my dog inappropriate food because I like to spoil him/her. | 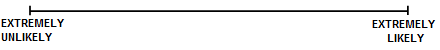 |
| 48. My dog is overfed because I indulge him/her. | 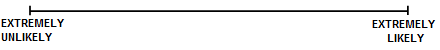 |
| 49. I feed my dog inappropriate food because appropriate food is too expensive. | 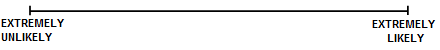 |
| 50. Overall, how much control do you feel you have over the amount you feed your dog? | 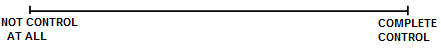 |
| 51. Overall, how much control do you feel you have over the type of food you feed your dog? | 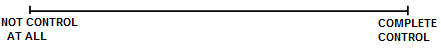 |
| 52. Overall, how much control do you feel you have over the number of times you feed your dog during the day? | 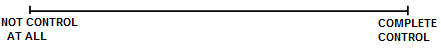 |
| 53. It’s important that I exercise my dog the appropriate number of times a week. | 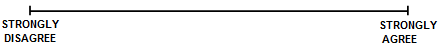 |
| 54. It's important that I give my dog the appropriate type of exercise. | 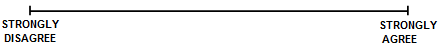 |
| 55. It’s important to me that my dog is fit. | 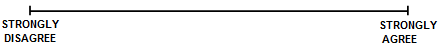 |
| 56. My dog doesn’t need exercise. | 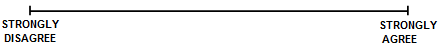 |
| 57. It's important that I exercise my dog for the appropriate length of time. | 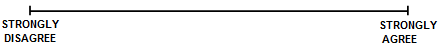 |
| 58. I don't know how often I should exercise my dog. | 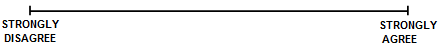 |
| 59. I don't know the appropriate length of time my dog should be exercised. | 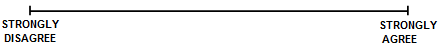 |
| 60. I don't know what type of exercise to give my dog. | 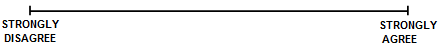 |
| 61. It's important that I exercise my dog as frequently as he/she wants. | 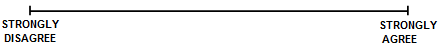 |
| 62. It's important that I exercise my dog for as long as he/she wants. | 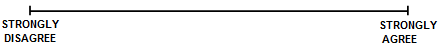 |
| 63. It's important that I give my dog the type of exercise that he/she likes. | 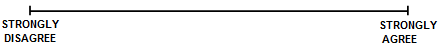 |
| 64. I don't exercise my dog frequently enough because I don't like to. | 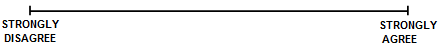 |
| 65. I don't exercise my dog for long enough because I don't like to. | 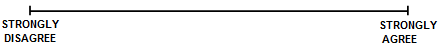 |
| 66. I don't give my dog the appropriate type of exercise because I don't like to. | 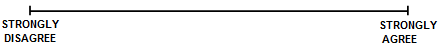 |
| 67. I don’t give my dog the appropriate kind of exercise because he/she doesn’t like that type of exercise. | 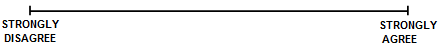 |
| 68. I don’t exercise my dog as frequently as I should because he/she is badly behaved. | 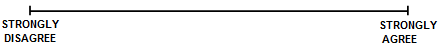 |
| 69. I don’t exercise my dog as frequently as I should because I don’t have time. | 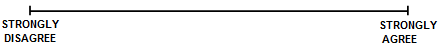 |
| 70. I don’t give my dog the appropriate type of exercise because I don’t have access to appropriate areas. | 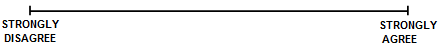 |
| 71. My dog isn’t exercised frequently enough because other people exercise it. | 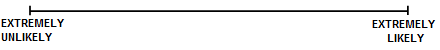 |
| 72. My dog isn't given the appropriate type of exercise because other people exercise it. | 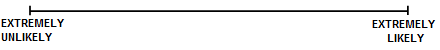 |
| 73. My dog isn't exercised long enough because other people exercise it. | 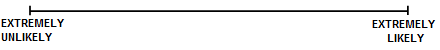 |
| 74. Overall, how much control do you feel you have over the type of exercise you give your dog? | 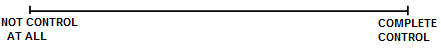 |
| 75. Overall, how much control do you feel you have over how frequently you exercise your dog? | 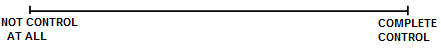 |
| 76. Overall, how much control do you feel you have over the length of time you exercise your dog? | 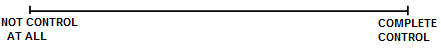 |

THANK YOU VERY MUCH for your participation.
